# Persistent Activation of Sphingosine‐1‐Phosphate Receptor 1 by Phytosphingosine‐3,4‐Cyclic Phosphate Ameliorates Sepsis by Inhibiting Hyperinflammation and Vascular Hyperpermeability

**DOI:** 10.1002/mco2.70238

**Published:** 2025-06-03

**Authors:** Suhong Duan, Seung‐Gook Kim, Jiaying Bao, Hyung‐Jin Lim, Joon Woo Kim, Sung‐Il Yoon, Young Jun Park, Sanuk Yun, Kye‐Seong Kim, Hwa‐Ryung Song, Myeong Jun Choi, Myung‐Kwan Han

**Affiliations:** ^1^ Department of Microbiology Jeonbuk National University Medical School Jeonju‐si Jeollabuk‐do Republic of Korea; ^2^ Department of Pediatrics Shandong Provincial Hospital Affiliated to Shandong First Medical University Jinan Shandong Province China; ^3^ Axceso Biopharma, 282 Hagui‐ro Yongin‐si Gyeonggi‐do Republic of Korea; ^4^ Department of Biotechnology Inje University Gimhae Republic of Korea; ^5^ Graduate School of Biomedical Science and Engineering Hanyang University Seongdong‐gu Seoul Republic of Korea

**Keywords:** cecal ligation and puncture, phytosphingosine‐3,4‐cyclic phosphate, sepsis, sirtuin 1 (SIRT1), sphingosine‐1‐phosphate, sphingosine‐1‐phosphate receptor 1

## Abstract

Sepsis is a life‐threatening disease characterized by multiorgan dysfunction caused by an abnormal immune response to microbial infection. Sphingosine‐1‐phosphate (S1P) levels are significantly lower in patients with sepsis and are negatively correlated with the severity of sepsis. However, whether the S1P signaling pathway is a target for sepsis treatment remains unknown. Here, we show that our newly synthesized phytosphingosine‐3,4‐cyclic phosphate (3,4‐cPP), a functional agonist of S1P receptor 1 (S1P1), exerts a strong protective effect against severe cecal ligation and puncture (CLP)‐induced sepsis. 3,4‐cPP persistently activates S1P1 without inducing internalization. 3,4‐cPP upregulates SIRT1 expression in macrophages and endothelial cells via S1P1 activation. Additionally, 3,4‐cPP decreases serum levels of proinflammatory cytokines, including IL‐6 and TNF‐α, and inhibits endothelial permeability in CLP‐induced septic mice. Conditional knockout of SIRT1, an NAD^+^‐dependent deacetylase, in macrophages or endothelial cells counteracts the inhibition of inflammatory cytokine secretion and prevention of endothelial cell permeability by 3,4‐cPP in CLP‐induced septic mice, indicating that the S1P1/SIRT1 axis in both the endothelium and macrophages is essential for survival in sepsis. Collectively, the data suggest that prolonged activation of the S1P1/SIRT1 signaling pathway protects against sepsis by inhibiting hyperinflammation and vascular hyperpermeability.

## Graphical abstract

Phytosphingosine‐3,4‐cyclic phosphate was synthetized from phytosphingosine. Phytosphingosine‐3,4‐cyclic phosphate had a strong therapeutic effect on sepsis through sphingosine‐1‐phosphate receptor 1 (S1P1)‐mediated inhibition of hyperinflammation in macrophages and vascular hyperpermeability in endothelium via SIRT1 upregulation.

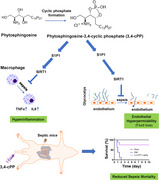

## 1 Introduction

Sepsis is a life‐threatening condition that arises from an abnormal immune response to microbial infection, leading to multiorgan failure with up to 30% mortality rate [1, 2]. Although sepsis treatments have traditionally focused on improving immune dysfunction, many attempts using anti‐inflammatory drugs have failed [3]. Given that increased vascular permeability and dysregulated immunity contribute to sepsis [4, 5, 6], addressing immune responses and vascular hyperpermeability is crucial in its treatment.

The glycocalyx is a carbohydrate‐rich, gel‐like layer on the luminal surface of vascular endothelial cells [7] that regulates vascular permeability, prevents microvascular thrombosis, and regulates leukocyte adhesion to the vascular endothelium [8, 9]. Heparan sulfate proteoglycans (HSPGs) are a major component of the glycocalyx, particularly in the endothelial glycocalyx [10]. HSPGs function as structural components and signaling molecules, contributing to the integrity of endothelial barrier, mechanotransduction, and ligand recognition [10]. During sepsis, inflammatory enzymes such as metalloproteinases, heparinases, and hyaluronidases disrupt this layer [11, 12, 13]. Inflammation‐induced glycocalyx disruption increases vascular permeability, dysregulates vasodilation and microvascular thrombosis, and enhances leukocyte adhesion [14]. Thus, endothelial dysfunction and glycocalyx degeneration are primary targets of sepsis therapy.

Sphingosine‐1‐phosphate (S1P), a signaling mediator produced by sphingosine kinase 1 (Sphk1) from sphingosine, participates in various physiological and pathological processes through interaction with five G protein‐coupled receptors (GPCRs): S1P receptor 1 (S1P1), S1P2, S1P3, S1P4, and S1P5 [15, 16]. S1P blocks glycocalyx shedding via S1P1 and maintains vascular homeostasis [17, 18]. S1P binding to S1P1 promotes protection of the endothelium and anti‐inflammatory responses during SARS‐CoV‐2 infection, whereas binding to S1P2 counteracts the effects of S1P1 [19], leading to vascular inflammation, endothelial permeability, and organ dysfunction. Additionally, S1P signaling of S1P1 promotes endothelial cell barrier stability, whereas S1P2 and S1P3 signaling enhance vascular permeability [20]. Therefore, developing novel S1P receptor agonists that can stimulate S1P1 without activating S1P2 or S1P3 is expected to aid the treatment of endothelial damage.

Serum S1P levels in patients with sepsis are markedly suppressed and inversely correlated with sepsis severity [21], suggesting that S1P supplement may elicit a positive effect. However, plasma S1P is rapidly cleaved by lipid phosphate phosphohydrolase 1 following intravenous injection into mice [22, 23]. Thus, many S1P1 agonists have been developed, including FYT720 (fingolimod), KRP203 (mocravimod), and SEW2871 [24, 25, 26], that functionally act as antagonists rather than agonists by decreasing membrane S1P1 expression via S1P receptor internalization. These compounds significantly inhibit S1P1‐dependent lymphocyte egress from secondary lymphoid organs and reduce the number of peripheral blood lymphocytes, contributing to their therapeutic effects against various autoimmune diseases. Hence, these agonists may not improve endothelial cell defense or exert anti‐inflammatory effects. Indeed, significant differences were not observed between patients with sepsis and moderate coronavirus disease 2019 (COVID‐19) treated with fingolimod, an immunomodulator used for multiple sclerosis, and the control group regarding intubation or mortality rate [27].

Silent information regulator 2 homolog 1 (SIRT1) is an NAD+‐dependent deacetylase that modifies the activity of target proteins and regulates their function by removing acetyl groups [28]. SIRT1 protects against lethal endotoxicity and septic shock via immunosuppression through nuclear factor‐kappa B (NF‐κB) deacetylation [29, 30]. Additionally, SIRT1 relieves sepsis by inhibiting glycocalyx degradation, thereby normalizing vascular permeability [31]. Meanwhile, blocking S1P production through Sphk1 knockdown inhibits SIRT1 expression [32], suggesting that S1P increases SIRT1 expression. Hence, SIRT1 is an essential target for sepsis treatment that can simultaneously inhibit immunosuppression and vascular permeability. Taken together, we hypothesize that S1P‐induced upregulation of SIRT1 mediates S1P‐induced enhancement of endothelial cell barrier stability.

In this study, we synthesized phytosphingosine‐3,4‐cyclic phosphate (3,4‐cPP) and investigated whether it modulates S1P signaling and alters endothelial cell permeability in a septic mouse model, ameliorating sepsis.

## 2 Results

### 2.1 3,4‐cPP is a Novel Long‐Acting S1P1 Agonist

3,4‐cPP was chemically synthesized through several reaction steps using phytosphingosine as the starting material. The characteristics of the synthesized 3,4‐cPP were confirmed by liquid chromatography with tandem mass spectrometry (LC–MS/MS) and nuclear magnetic resonance (NMR) spectroscopy (Figures S1A,B). Unlike S1P and phytosphingosine‐1‐phosphate (P1P), which have a monophosphate group attached, 3,4‐cPP has a cyclic diphosphate group that is less likely to be attacked by S1P phosphatase and lipid phosphate phosphatase in the plasma. Although S1P has a half‐life of 15 min [22, 23], 3,4‐cPP was plasma‐stable, with >90% in human plasma and > 88% in mouse plasma after 180 min (Figure S1C). 3,4‐cPP half‐life elimination (*t*1/2) was approximately 1.5 h following intravenous administration of 100 µg/kg to mice. The structural similarities between 3,4‐cPP and S1P and 3,4‐cPP's relative stability in blood suggest its potential as a functional agonist for S1P receptors.

**FIGURE 1:**
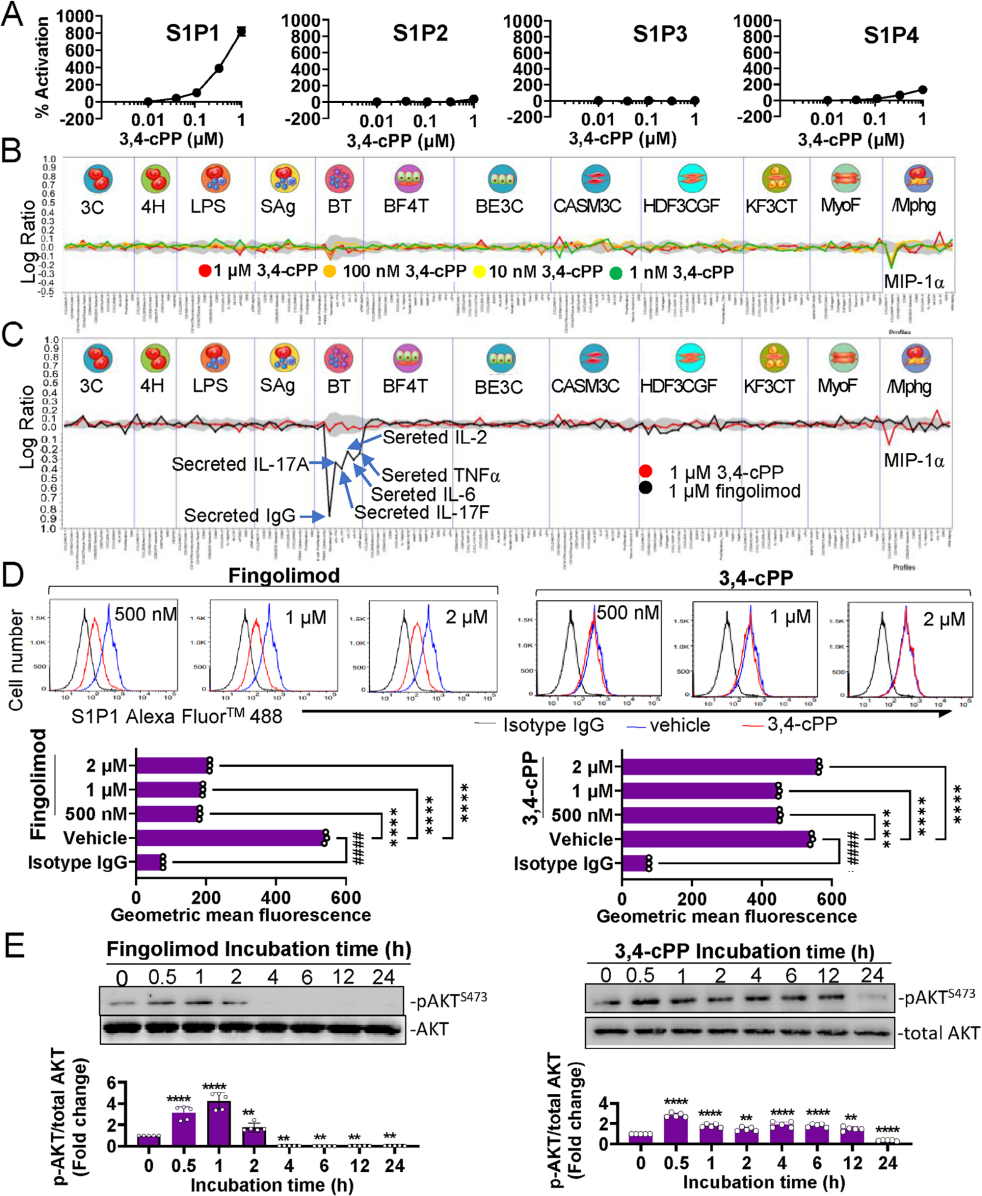
3,4‐cPP is a long‐acting S1P1 agonist. (A) Dose–response curves of PathHunter β‐arrestin recruitment to S1P1, S1P2, S1P3, and S1P4 by 3,4‐cPP treatment. Values represent % activation in the basal enzyme fragment complementation signal (mean ± SD, *n* = 4). (B) Biological effects of 3,4‐cPP in primary human cell‐based models. 3,4‐cPP was tested in 12 BioMAP systems (3C, 4H, LPS, Sag, BT, BF4T, BE3C, CASM3C, HDF3CGF, KF3CT, MyoF, and /Mphg; see Figure S2 for additional description) at concentrations of 1 µM (green), 100 nM (orange), 10 nM (yellow), and 1 nM (red). The 12 BioMAP systems are divided by vertical lines and feature representative icons at the top. The measured biomarkers are displayed on *x*‐axis. The *y*‐axis represents the log expression ratios of the readout biomarker levels treated with 3,4‐cPP in relation to vehicle controls. Values outside the 95% confidence interval (gray region) significantly differed from those of vehicle controls. (C) The comparison of biological effects of 3,4‐cPP and fingolimod. 3,4‐cPP and fingolidmod were tested 12 BioMAP systems at a concentration of 1 µM. Common biomarker readouts were annotated when the readings for both profiles fell within the significance range, showing an effect size greater than 20% (|log10 ratio| > 0.1) in the same direction. (D) Histogram (upper panels) and mean fluorescence analysis (lower panels) of cell surface S1P1 in RAW cells exposed to 500 nM–2 µM fingolimod and 3,4‐cPP for 90 min. (E) Time‐dependent phosphorylation of AKT in RAW cells exposed to 500 nM fingolimod (left panel) and 500 nM 3,4‐cPP (right panel). Protein bands were quantified using densitometric analysis, where pAKT levels were normalized to total Akt levels (lower panels). The signals of AKT and pAKT were quantitated using the NIH Image analysis program, and the ratios of phosphorylated AKT to total AKT are shown in the bar diagram (mean ± SD, *n* = 5). The ratio of phosphorylated AKT to total AKT in untreated cells (time 0) was set as 1. *p < 0.05, **p < 0.01, ***p < 0.001, ****p < 0.0001 versus 0 h incubation (one‐way ANOVA and Turkey's multiple comparisons). S1P1–5, sphingosine‐1‐phosphate receptor 1–5; 3,4‐cPP, phytosphingosine‐3,4‐cyclic phosphate; MIPα, macrophage inhibitory protein 1 alpha.

The GPCR cell‐based agonist arrestin biosensor assay demonstrated that 3,4‐cPP exhibited high binding activity with S1P1 and negligible binding with S1P2, S1P3, and S1P4 (Figure 1A). Having demonstrated the ability of 3,4‐cPP to bind and activate S1P1 in the designed cellular assays, 3,4‐cPP was tested for direct human cell‐based biological effects in a panel of complex primary human cell systems, namely, BioMAP systems (Figure S2), to define the mechanisms of action and its potential to cause off‐target effects. This panel comprised 148 biological markers across 12 primary human cell models, representing various aspects of human tissue biology and disease (Figure S2). When tested at 1 nM to 1 µM, 3,4‐cPP showed no cytotoxic effects (Figure 1B). Treatment with 1 µM 3,4‐cPP inhibited the production of macrophage inhibitory protein 1 alpha, which belongs to the C‐C subgroup of chemokines and exhibits several proinflammatory roles, including immune cell chemotaxis, indicating that 3,4‐cPP did not significantly affect the other 11 BioMAP models (Figure 1B). However, 3,4‐cPP showed no significant effect on the 11 other BioMAP models except/Mphg model (Figure 1B), indicating that 3,4‐cPP exerted minimal or weak off‐target effects. In contrast, fingolimod inhibited the production of IgG, IL‐17A, IL‐17F, IL‐6, and TNF‐α. A comparison of the 3,4‐cPP and fingolimod profiles revealed different activities with no common activity (Figure 1C). Fingolimod functionally acts as an antagonist of lymphocytic S1P1 and inhibits lymphocyte egress from secondary lymphoid organs by long‐term internalization and degradation of S1P1 [33, 34]. We found that 3,4‐cPP negligibly downregulated cell surface S1P1, whereas FTY720 downregulated 77% of cell surface S1P1 in RAW 264.7 cells (Figure 1D, lower panels), indicating that 3,4‐cPP persistently activates S1P1 without internalizing S1P1. AKT phosphorylation, a target of the S1P1 downstream pathway, was activated by 3,4‐cPP for longer than by fingolimod (Figure 1E). These results suggest that 3,4‐cPP is a novel long‐acting functional S1P1 agonist with a mechanism of action different from that of fingolimod.

**FIGURE 2:**
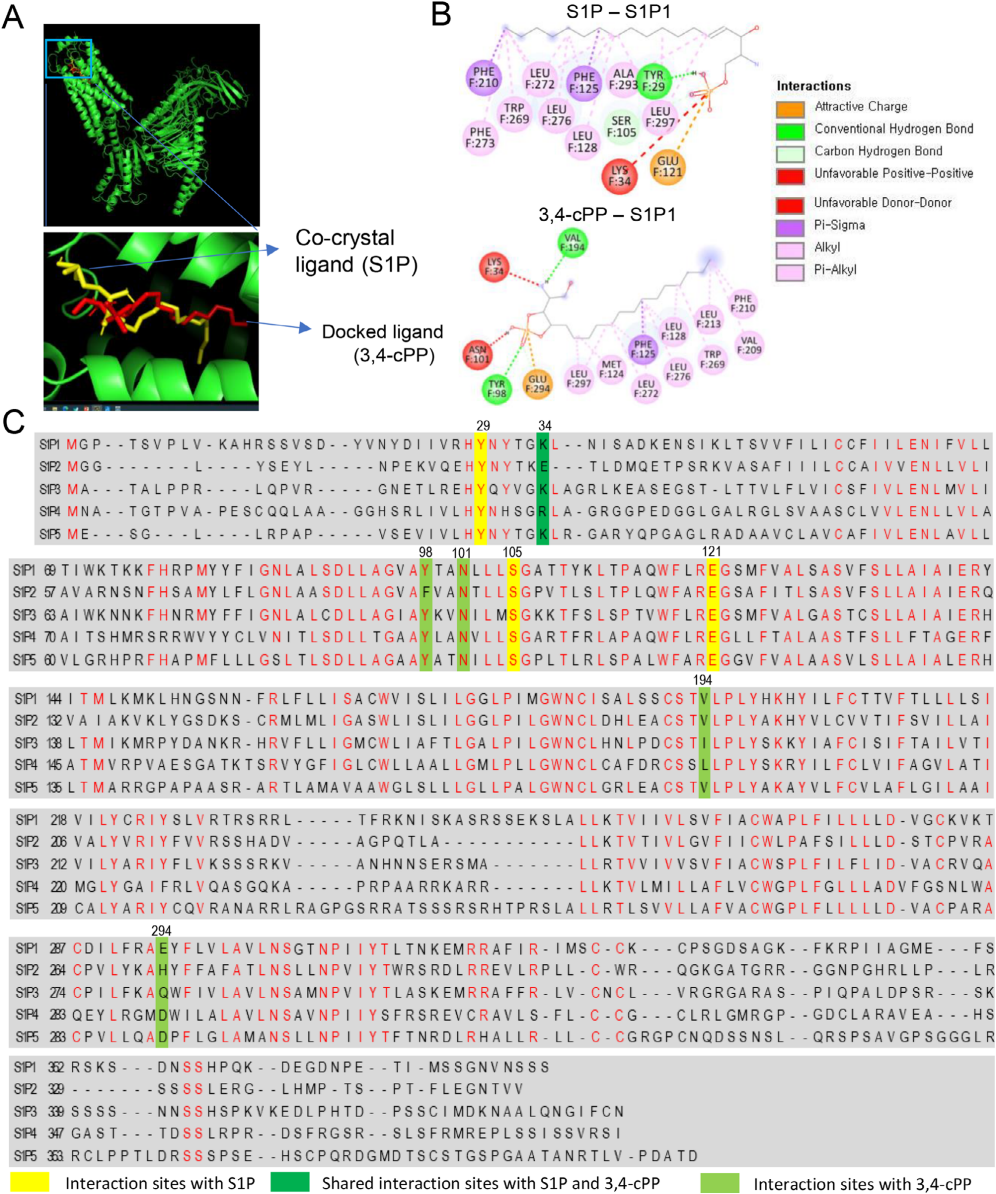
(A) 3D interaction of S1P and 3,4‐cPP with S1P1 docked using AutoDock Vina and visualized using Discovery Studio. The docking site shows superimposed conformations of the co‐crystallized S1P (yellow) and the docked ligand 3,4‐cPP (red). (B) 2D ligand interactions of S1P and 3,4‐cPP with the active site of S1P1. (C) Multiple sequence alignment analysis of S1P1–S1P5. Residues conserved among the S1P receptor sequences are highlighted in red. Nine amino acid residues involved in attractive charge, hydrogen bond, and unfavorable interactions except for hydrophobic interactions between S1P and S1P1 or 3,4‐cPP and S1P1 are indicated as yellow boxes (interaction with S1P), dark green boxes (shared interaction with S1P and 3,4‐cPP) and light green boxes (interaction with 3,4‐cPP). S1P, sphingosine‐1‐phosphate; S1P1, sphingosine‐1‐phosphate receptor 1; 3,4‐cPP, phytosphingosine‐3,4‐cyclic phosphate.

Molecular docking studies were performed using AutoDock Vina to visualize the binding interactions of S1P and 3,4‐cPP with S1P1 (Figure 2A,B). S1P formed one unfavorable bond with Lys34 in S1P1 while 3,4‐cPP formed two unfavorable bonds with Lys34 and Asn101 in S1P1 (Figure 2B). The highest scoring ligand for 3,4‐cPP had a binding affinity score of ‐7.1 using AutoDock Vina, compared with S1P (−6.5). S1P and 3,4‐cPP formed favorable attractive charge interactions between the phosphate group of S1P and Glu121 in S1P1 and between the phosphate group of 3,4‐cPP and Glu294 in S1P1 (Figure 2B). S1P formed conventional or carbon hydrogen bonds with Tyr29 and Ser105 in S1P1, whereas 3,4‐cPP formed bonds with Tyr98 and Val194 in S1P1 (Figure 2B). S1P also formed numerous hydrophobic interactions, including pi‐sigma, alkyl, and pi‐alkyl interactions with Phe125, Leu128, Phe210, Trp269, Leu272, Phe273, Leu276, Ala293, and Leu297 in S1P1 (Figure 2B). 3,4‐cPP also formed numerous hydrophobic interactions with Met124, Phe125, Leu128, Val209, Phe210, Leu213, Trp269, Leu272, Leu276, and Leu297 in S1P1 (Figure 2B).

When the amino acid sequences of S1P1–S1P5 were aligned, three amino acid residues (Tyr29, Ser105, and Glu121 in S1P1) involved in attractive charges, hydrogen bonding, and unfavorable interactions, excluding hydrophobic interactions between S1P and S1P1, were aligned with those in other S1P receptors (Figure 2C). However, one residue involved in an unfavorable bond (Lys34 in S1P1) was not aligned with the same residue in other S1P receptors, indicating that the residues aligned with Lys34 of S1P1 in other S1P receptors confer unique affinities of S1P and S1P receptors (Figure 2C). However, four amino acid residues (Lys34, Tyr98, Val194, and Glu294 in S1P1) involved in attractive charges, hydrogen bonding, and unfavorable interactions, excluding hydrophobic interactions, between S1P and 3,4‐cPP, were not aligned with those in other S1P receptors (Figure 2C). One residue (Asn101 in S1P1) involved in unfavorable interactions was aligned with the same residue in other S1P receptors (Figure 2C). The variability in amino acid residues among S1P receptors interacting with 3,4‐cPP likely confers selectivity to 3,4‐cPP for S1P1.

Our data indicate that 3,4‐cPP is a functional agonist that selectively activates S1P1 for a relatively longer duration than fingolimod.

### 2.2 3,4‐cPP Upregulates SIRT1 Expression in Macrophages and Endothelial Cells

**FIGURE 3:**
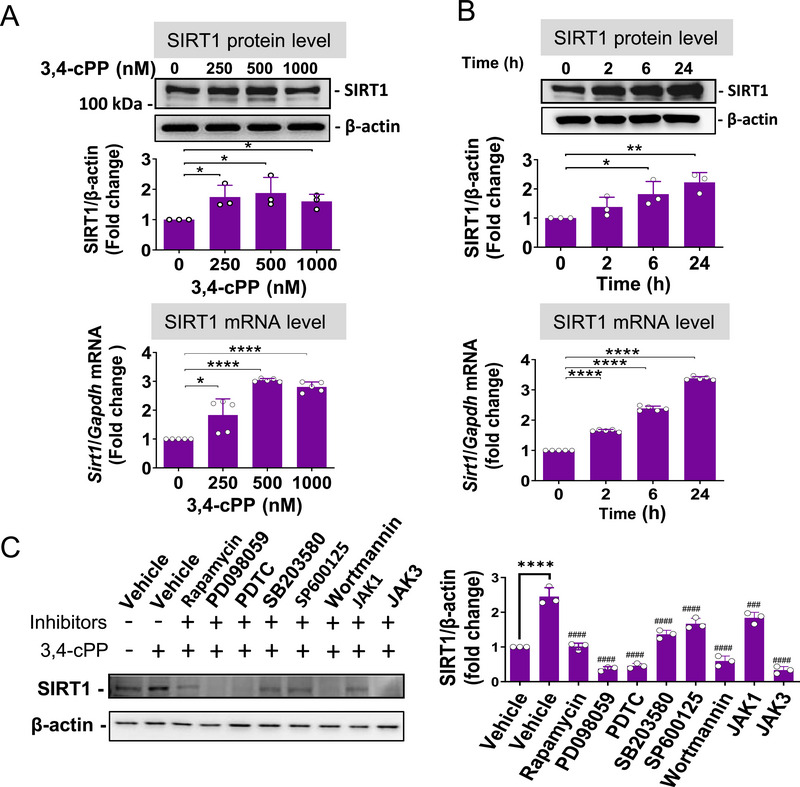
The effect of 3,4‐cPP on the expression of SIRT1 in RAW cells. (A and B) Concentration‐ (A) and time‐ (B) dependent expression of SIRT1 protein (upper panels) and mRNA (lower panels) by the treatment of 3,4‐cPP in RAW cells. RAW cells were treated with 0–1000 nM 3,4‐cPP for 24 h (A) or 500 nM 3,4‐cPP for 0–24 h (B). Representative Western blotting results are shown in the upper panels. Densitometric data are shown as mean ± SD (*n* = 3). mRNA expression levels were quantified using RT‐PCR and normalized to *Gapdh* (mean ± SD, *n* = 5). *p < 0.05, **p < 0.01, ***p < 0.001, ****p < 0.0001 versus control (one‐way ANOVA and Turkey's multiple comparison). (C) The effects of signal transduction inhibitors on 3,4‐cPP‐induced SIRT1 expression. RAW cells were pretreated with 10 µM rapamycin, 10 µM PD098059, 10 µM PDTC, 10 µM SB203580, 10 µM SP600125, 1 µM wortmannin, 5 µM LY294002, 5 µM JAK1 inhibitor (JAK1), or 10 µM JAK3 inhibitor (JAK3) for 1 h and followed by treatment with 500 nM 3,4‐cPP for 24 h. Representative protein bands (left panel) and quantitative Western blot analyses of SIRT1 (right panel) (mean ± SD, *n* = 5) are presented. ****p < 0.0001 versus control and ^####^ *p* < 0.0001 versus 3,4‐cPP treatment alone (one‐way ANOVA and Turkey's multiple comparison). 3,4‐cPP, phytosphingosine‐3,4‐cyclic phosphate; *Gapdh*, glyceraldehyde 3‐phosphate dehydrogenase; RT‐PCR, real‐time polymerase chain reaction; PDTC, Ammonium pyrrolidine dithiocarbamate; JAK, Janus kinase.

BioMAP phenotypic screening assay results indicated that 3,4‐cPP exhibited anti‐inflammatory activity in macrophages (Figure 1B). SPHK/S1P signaling upregulates SIRT1 expression in leukemia cells [32]. Interferon β induces SIRT1 expression in macrophages [30], alleviating sepsis by inhibiting proinflammatory cytokines. Accordingly, we speculated that S1P alleviates sepsis by inducing SIRT1 expression in macrophages. 3,4‐cPP enhanced SIRT1 expression in a dose‐dependent manner in RAW 246.7 macrophages, with maximum increase in SIRT1 protein and mRNA expression at 500 nM (Figure 3A). Additionally, 500 nM 3,4‐cPP increased SIRT1 protein and mRNA expression in RAW macrophages in a time‐dependent manner, with maximum expression observed at 24 h (Figure 3B). In contrast, fingolimod slightly decreased SIRT1 expression at 1 µM (Figure S3).

To determine whether 3,4‐cPP induces SIRT1 expression through S1P1, we investigated the effect of S1P1 knockdown on 3,4‐cPP‐induced SIRT1 expression in RAW macrophages. The knockdown of SIP1 using siRNA abolished 3,4‐cPP‐induced increase in SIRT1 expression in RAW cells (Figure S4A). These results indicate that persistent activation of S1P1 by 3,4‐cPP induces SIRT1 expression in RAW macrophages. Moreover, treatment of these cells with inhibitors of mammalian target of rapamycin (mTOR; i.e., rapamycin), extracellular signal‐regulated kinase (ERK; i.e., PD098059), NF‐κB (ammonium pyrrolidine dithiocarbamate [PDTC]), phosphoinositide 3‐kinase (PI3K; i.e., wortmannin), and Janus kinase 3 (JAK3) abrogated 3,4‐cPP‐induced SIRT1 expression (Figure 3C). Hence, 3,4‐cPP‐induced SIRT1 expression in macrophages requires activation of multiple signaling pathways, including mTOR, ERK, NF‐κB, PI3K, and JAK–signal transducer and activator of transcription (STAT).

**FIGURE 4:**
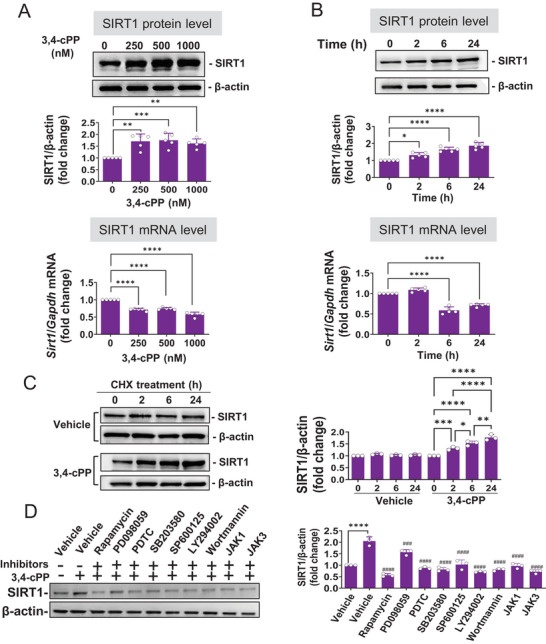
The effect of 3,4‐cPP on the expression of SIRT1 in MYSECs. (A and B) Concentration‐ (A) and time‐ (B) dependent expression of SIRT1 protein (upper panels) and mRNA (lower panels) by the treatment of 3,4‐cPP in MYSECs. MYSECs were incubated with 0–1000 nM 3,4‐cPP for 24 h (A) or 500 nM 3,4‐cPP for 0–24 h (B). Representative Western blotting results are shown in the upper panels. The densitometric data are shown as mean ± SD (*n* = 5). mRNA expression levels were quantified using RT‐PCR and normalized to *Gapdh* (mean ± SD, *n* = 5). *p < 0.05, **p < 0.01, ***p < 0.001, ****p < 0.0001 vs. control (one‐way ANOVA and Turkey's multiple comparison). (C) Upregulation of SIRT1 expression by 3,4‐cPP in a protein synthesis‐independent manner in MYSECs. MYSECs were incubated with or without 500 nM 3,4‐cPP for 24 h in the presence of 5 µM cycloheximide (CHX). Equal aliquots of cells were collected at 0, 2, 6, and 24 h after CHX treatment for western blot analysis. Representative protein bands (left panel) and quantitative western blot analysis (right panel) are shown (mean ± SD, *n* = 3). *p < 0.05, **p < 0.01, ***p < 0.001 versus 3,4‐cPP treatment at 0 h (one‐way ANOVA and Turkey's multiple comparison). (D) The effects of signal transduction inhibitors on 3,4‐cPP‐induced SIRT1 expression in MYSECs. MYSECs were pretreated with 10 µM rapamycin, 10 µM PD098059, 10 µM PDTC, 10 µM SB203580, 10 µM SP600125, 1 µM wortmannin, 5 µM JAK1 inhibitor (JAK1), or 10 µM JAK3 inhibitor (JAK3) for 1 h and followed by treatment with 500 nM 3,4‐cPP for 24 h. Representative protein bands (upper panel) and quantitative western blot analysis of SIRT1 (lower panel) (mean ± SD, *n* = 3) are presented. ****p < 0.0001 versus control and ^###^ *p *< 0.001, ^####^ *p* < 0.0001 versus 3,4‐cPP treatment alone (one‐way ANOVA and Turkey's multiple comparison). 3,4‐cPP, phytosphingosine‐3,4‐cyclic phosphate; *Gapdh*, glyceraldehyde 3‐phosphate dehydrogenase; CHX, cycloheximide; JAK, Janus kinase.

To determine whether 3,4‐cPP affects SIRT1 expression in endothelial cells, another central sepsis target, we assessed the effect of 3,4‐cPP using murine yolk sac endothelial cells (MYSECs). 3,4‐cPP increased SIRT1 protein in MYSECs in a dose‐dependent manner, with maximum increase at 500 nM (Figure 4A, upper panel). Additionally, 500 nM 3,4‐cPP enhanced SIRT1 protein levels in a time‐dependent manner, peaking at 24 h (Figure 4B, upper panel). Although 3,4‐cPP increased SIRT1 protein levels, it decreased mRNA levels in MYSECs (Figure 4A,B, lower panels). SIRT1 protein expression was increased by 3,4‐cPP even in the presence of cycloheximide, a protein synthesis inhibitor (Figure 4C). Thus, 3,4‐cPP‐induced upregulation of SIRT1 in endothelial cells does not proceed through *Sirt1* transcription, but by inhibiting SIRT1 protein degradation.

The knockdown of SIP1 using siRNA abolished 3,4‐cPP‐induced upregulation of SIRT1 in MYSECs (Figure S4B), suggesting that 3,4‐cPP increased SIRT1 expression via S1P1. Rapamycin, PDTC, SB203580, SP600125, LY294002 (a PI‐3K inhibitor), wortmannin, a JAK1 inhibitor, and a JAK3 inhibitor abrogated 3,4‐cPP‐induced SIRT1 expression (Figure 4D). These results suggest that 3,4‐cPP‐induced SIRT1 expression in endothelial cells requires activation of multiple signaling pathways, including mTOR, p38, JNK, PI3K, and JAK–STAT. The signaling pathways required for SIRT1 expression in endothelial cells differ slightly from those in macrophages. These differences likely led to the distinct mode of SIRT1 expression mediated by 3,4‐cPP in macrophages and endothelial cells. Cumulatively, these data indicate that 3,4‐cPP upregulates SIRT1 expression by activating S1P1 in macrophages and endothelial cells.

### 2.3 Intravenous Injection of 3,4‐cPP Protects Against Sepsis by Upregulating SIRT1 in Macrophages and Endothelial Cells

**FIGURE 5:**
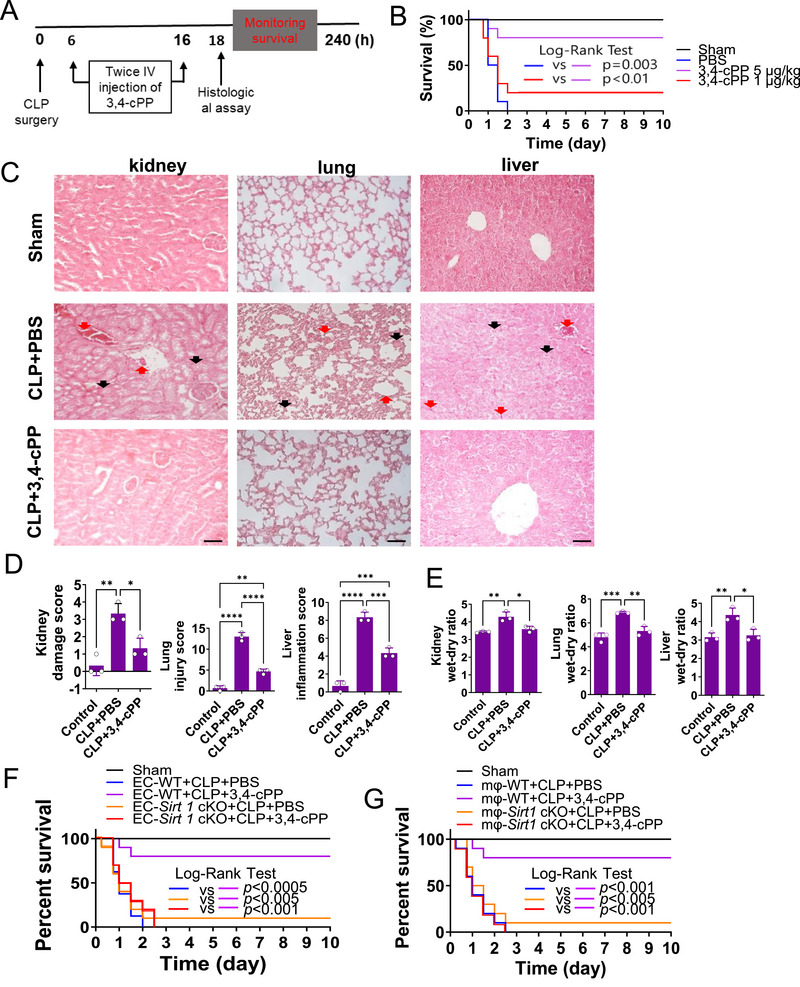
Intravenous administration of 3,4‐cPP mitigates cecal ligation and puncture (CLP)‐induced sepsis by increasing SIRT1 expression in macrophages and endothelial cells. (A) Illustration of experimental design. (B) C57BL/6 mice underwent moderate CLP. Intravenous injections of 3,4‐cPP (5 µg/kg) were administered at 6 and 16 h after CLP surgery. The survival was monitored for 10 days (*n* = 40 mice/group). (C) Representative H&E images of kidney, lung, and liver sections from sham‐operated or septic mice treated with PBS or 3,4‐cPP (×200 magnification). Eighteen hours after CLP surgery, lung, liver, and kidney tissues were collected and stained with H&E. Black arrows in the kidney indicate proximal convoluted tubule swelling and red arrows in the kidney indicate inflammatory cell infiltration. Black arrows in the lung indicate pulmonary edema and red arrows in the lung indicate inflammatory cell infiltration. Black arrows in the liver indicate hepatocellular edema and red arrows in the liver indicate inflammatory cell infiltration. (D) The effect of 3,4‐cPP on the histological injury scores in the kidney, lung, and liver tissues of sham‐operated or septic mice treated with PBS or 3,4‐cPP. Histological injury scores were quantified as described in *Materials and Methods* (mean ± SD, *n* = 3). Data are the average of 3 mice per group, with three sections scanned per organ, three random sites per sections and values averaged to produce one value for each organ. *p < 0.05, **p < 0.01, ***p < 0.001, ****p < 0.0001 (one‐way ANOVA and Turkey's multiple comparison). (E) The wet‐to‐dry weight ratios of the kidneys, lungs, and livers of sham‐operated and septic mice treated with PBS or 3,4‐cPP (mean ± SD, *n* = 3). *p < 0.05, **p < 0.01, ***p < 0.001 (one‐way ANOVA and Turkey's multiple comparison). (F and G) The effect of EC–*Sirt1* cKO (F) and MФ–*Sirt1* cKO (G) on 3,4‐cPP‐mediated protection against CLP‐induced sepsis. EC–*Sirt1* cKO and MФ–*Sirt1* cKO mice underwent moderate CLP. Intravenous injections of 3,4‐cPP (5 µg/kg) were administered at 6 and 16 h after CLP surgery. Survival was monitored for 10 days (*n* = 10 mice/group). CLP, cecal ligation and puncture; IV, intravenous; 3,4‐cPP, phytosphingosine‐3,4‐cyclic phosphate; PBS, phosphate‐buffered saline; EC‐WT, endothelial cell wild type; mФ‐WT, macrophage wild type; EC–*Sirt1* cKO, endothelial cell‐specific *Sirt1* conditional knockout; MФ–*Sirt1* cKO, macrophage‐specific *Sirt1* conditional knockout.

To determine the effect of 3,4‐cPP on highly inflammatory sepsis, we administered 3,4‐cPP to cecal ligation and puncture (CLP)‐induced sepsis mice and monitored their survival for over 10 days. Intravenous injection of 3,4‐cPP (5 µg/mouse) at 6 and 16 h post‐CLP surgery increased survival rates by 80% on Day 10 compared with vehicle injection (Figure 5A,B; log‐rank test, *p* = 0.003). These results indicate that 3,4‐cPP was highly effective against sepsis, even in a severe CLP sepsis model in which all animals died within 48 h. Notably, 3,4‐cPP was highly protective against sepsis even when administered 6 h after sepsis induction. However. fingolimod showed no protective effect against sepsis at the same dose as 3,4‐cPP (Figure S5). This suggests that 3,4‐cPP and fingolimod have different biological effects.

To examine the effect of 3,4‐cPP on organ damage induced by CLP, lung, liver, and kidney samples were excised and histologically examined (Figure 5C). 3,4‐cPP increased SIRT1 expression in the organs of septic mice, including the lungs and the liver (Figure S6). CLP increased kidney, lung, and liver damage scores compared with sham (Figure 5D). 3,4‐cPP administration abolished CLP‐induced increase in organ damage scores (Figure 5D). CLP significantly increased organ wet‐to‐dry weight ratios, which indicates edema, compared with sham (Figure 5E). 3,4‐cPP significantly decreased CLP‐induced rise in organ wet‐to‐dry weight ratios (Figure 5E). These results indicate that 3,4‐cPP reduces CLP‐induced septic death and multiorgan damage.

To determine whether 3,4‐cPP‐mediated SIRT1 expression in macrophages or endothelial cells is essential for its protective effect against sepsis, we created mice with endothelial cell‐specific *Sirt1* conditional knockout (EC–*Sirt1* cKO) and macrophage‐specific *Sirt1* cKO (MФ–*Sirt1* cKO), as previously described [31, 35]. The protective effect of 3,4‐cPP on CLP‐induced sepsis was abolished in either EC–*Sirt1* or MФ–*Sirt1* cKO (Figure 5F,G). These results indicate that endothelial and macrophage SIRT1 are essential for 3,4‐cPP‐mediated protection against sepsis. Furthermore, regulating inflammation and strengthening the integrity of the endothelial cell barrier are crucial for treating sepsis.

### 2.4 3,4‐cPP Suppresses the Expression of Inflammatory Cytokines in CLP‐Induced Sepsis Mice and Lipopolysaccharide‐Treated RAW Cells and MYSECs via SIRT1

**FIGURE 6:**
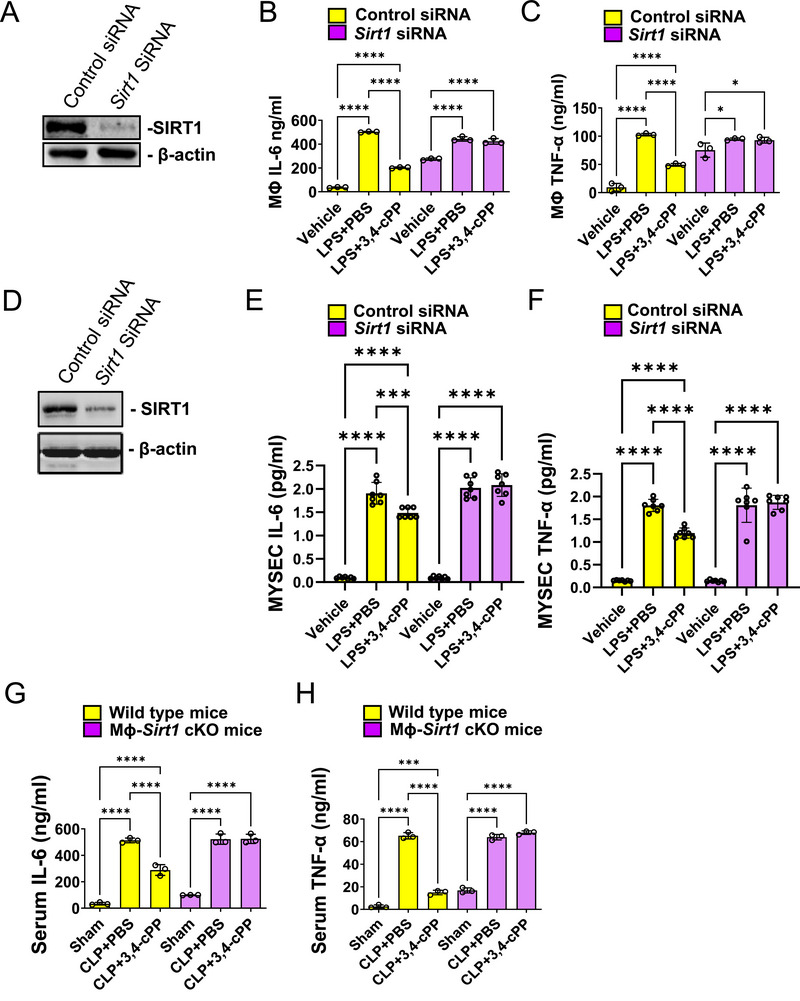
3,4‐cPP inhibits inflammatory cytokines in lipopolysaccharide (LPS)‐treated RAW cells and septic mice via SIRT1. (A) siRNA‐mediated knockdown of SIRT1 in RAW cells. RAW cells were transfected with siRNA targeting *Sirt1* (*Sirt1* siRNA) or negative control siRNA (control siRNA). The cells were harvested 24 h posttransfection and the lysates were subjected to western blotting. (B and C) IL‐6 (B) and TNF‐α (C) concentrations in the culture supernatant of control siRNA and *Sirt1* siRNA‐transfected RAW cells stimulated with or without LPS in the presence of 500 nM 3,4‐cPP. The content of IL‐6 and TNF‐α in the cell supernatants were assessed by ELISA (mean ± SD, *n* = 3). (D) siRNA‐mediated knockdown of SIRT1 in MYSECs. MYSECs were transfected with *Sirt1* or a negative control siRNA. The cells were harvested 24 h after transfection and the cell lysates were subjected to western blotting. (E and F) IL‐6 (E) and TNF‐α (F) concentrations in the culture supernatants of control siRNA‐ and *Sirt1* siRNA‐transfected RAW cells stimulated with or without LPS in the presence of 500 nM 3,4‐cPP. The content of IL‐6 and TNF‐α in the cell supernatant were assessed by ELISA (mean ± SD, *n* = 5). (G and H) IL‐6 (G) and TNF‐α (H) concentrations in the sera of Mϕ–*Sirt1* cKO and WT septic mice with or without 3,4‐cPP injection. Blood samples were collected at the indicated time points 18 h after CLP. IL‐6 and TNF‐α concentrations (B, C, E, F, G, and H) were measured using ELISA (mean ± SD, *n* = 3). *p < 0.05, **p < 0.01, ***p < 0.001, ****p < 0.0001 (one‐way ANOVA and Turkey's multiple comparison). LPS, lipopolysaccharide; PBS, phosphate‐buffered saline; 3,4‐cPP, phytosphingosine‐3,4‐cyclic phosphate; MФ–*Sirt1* cKO, macrophage‐specific *Sirt1* conditional knockout.

To investigate whether SIRT1 inhibits proinflammatory cytokine secretion, the effect of SIRT1 knockdown on lipopolysaccharide (LPS)‐induced secretion of IL‐6 and TNF‐α was evaluated in RAW cells (Figure 6A–C) and MYSECs (Figure 6D–F). SIRT1 knockdown abolished these inhibitory effects.

We also examined the effect of SIRT1 knockout on CLP‐induced secretion of IL‐6 and TNF‐α in mice. 3,4‐cPP decreased CLP‐induced IL‐6 and TNF‐α levels in wild‐type (WT) mice. In contrast, Mϕ–*Sirt1* cKO abolished this inhibitory effect of 3,4‐cPP (Figure 6G,H). These results suggest that 3,4‐cPP suppresses the secretion of proinflammatory cytokines in macrophages and endothelial cells by inducing SIRT1 expression, thereby alleviating sepsis.

### 2.5 3,4‐cPP Reverses Sepsis‐Induced Endothelial Damage via SIRT1 Expression

**FIGURE 7:**
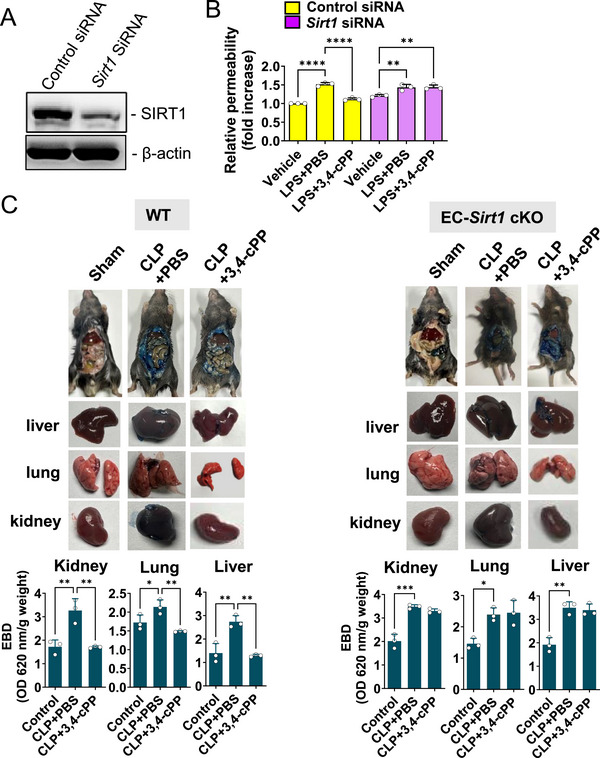
3,4‐cPP reverses endothelial damage induced by sepsis via SIRT1. (A) siRNA‐mediated knockdown of SIRT1 in MYSECs following transfection with negative control siRNA or *Sirt1* siRNA. (B) Reversal of the inhibitory action of 3,4‐cPP on LPS‐induced MYSEC hyperpermeability following *Sirt1* knockdown. MYSECs were grown on Transwell inserts at 300 ng/mL lipopolysaccharide (LPS) in the presence or absence of 500 nM 3,4‐cPP for 24 h. Streptavidin–peroxidase was added to the upper well and the medium was collected from the lower well after 5 min. Streptavidin–peroxidase flux was assessed by measuring absorbance at 530 nm after adding H_2_O_2_ and tetramethylbenzidine. Relative permeability was calculated and represented as fold‐difference relative to control (mean ± SD, *n* = 3). (C) The effect of 3,4‐cPP on CLP‐induced vascular permeability in WT (left panel) and EC–*Sirt1* cKO (right panel) mice. 3,4‐cPP (5 µg/mouse) was intravenously injected at 6 and 16 h after CLP operation. Eighteen hours after the operation, the mice received an injection of 0.5% EBD (200 µL/mouse) via the caudal vein. After 1 h, the organs of the mice were collected and imaged (upper panels), and EBD was extracted by incubating the samples with formamide at 55°C for 48 h. The EBD levels in the tissues was calculated by measuring absorbance at 620 nm with a 740 nm reference (mean ± SD, *n* = 3) (lower panels). *p < 0.05, **p < 0.01, ***p < 0.001, ****p < 0.0001 (one‐way ANOVA and Turkey's multiple comparison). LPS, lipopolysaccharide; PBS, phosphate‐buffered saline; 3,4‐cPP, phytosphingosine‐3,4‐cyclic phosphate; EC–*Sirt1* cKO, endothelial cell‐specific *Sirt1* conditional knockout.

SIRT1 mediates protection against endothelial damage during sepsis and endotoxemia [31, 36]. This suggests that 3,4‐cPP also affects sepsis‐induced endothelial damage by inducing SIRT1 expression. We first conducted a transwell permeability assay to evaluate the effect of 3,4‐cPP on LPS‐induced MYSEC permeability in vitro. 3,4‐cPP treatment significantly inhibited LPS‐induced increase in MYSEC permeability (Figure 7A,B). Meanwhile, SIRT1 knockdown abolished the inhibitory effects (Figure 7A,B). These results suggest that 3,4‐cPP protects against endothelial damage via SIRT1 upregulation.

To investigate whether 3,4‐cPP blocks sepsis‐induced increase in endothelial permeability, we examined its effect on CLP‐induced endothelial hyperpermeability using Evans blue dye (EBD). Compared with vehicle in WT mice, 3,4‐cPP significantly decreased EBD infiltration increase caused by CLP in the kidneys, lungs, and liver (Figure 7C, left). However, EC–*Sirt1* cKO eliminated the ability of 3,4‐cPP to reduce CLP‐induced infiltration of EBD into the kidneys, lungs, and liver (Figure 7C, right). These results suggest that 3,4‐cPP mitigates sepsis through SIRT1‐mediated inhibition of endothelial hyperpermeability.

**FIGURE 8:**
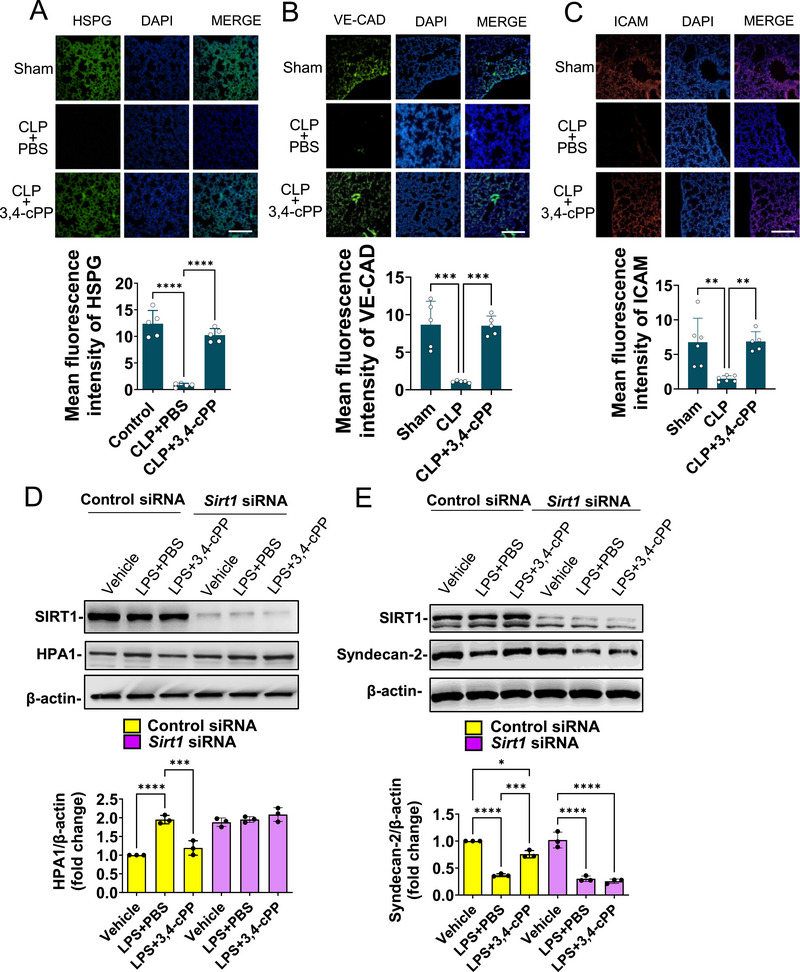
3,4‐cPP restores endothelial glycocalyx damage induced by CLP by regulating the SIRT1/HPA 1 pathway. (A–C) HSPG (A), VE‐cad (B), and ICAM (C) expression in lungs treated with PBS or 3,4‐cPP. 3,4‐cPP (5 µg/mouse) was intravenously injected at 6 and 16 h after CLP surgery. At 18 h after the surgery, HSPG, VE‐cad, and ICAM expression is shown by immunofluorescence (upper panels) and quantified as mean fluorescence intensity (mean ± SD, *n* = 5) (lower panels). Cell nuclei were stained with DAPI (blue). Scale bar: 200 µm. (D) Knockdown of SIRT1 eliminates the inhibitory effect of 3,4‐cPP on the LPS‐induced increase in HPA1 expression in MYSECs. Protein expression was analyzed using western blotting. Representative protein bands (upper panel) and quantitative western blot analysis of HPA1 (lower panel) are presented (mean ± SD, *n* = 3). (E) Knockdown of SIRT1 eliminates the restorative effect of 3,4‐cPP on LPS‐induced decrease in syndecan‐2 expression in MYSECs cultured under shear stress. Protein expression was analyzed using western blotting. Representative protein bands (upper panel) and quantitative Western blot analysis of syndecan‐2 (lower panel) are presented (mean ± SD, *n* = 3). MYSECs were transfected with control or *Sirt1* siRNA for 48 h and subsequently cultured under shear stress with 300 ng/mL LPS in the presence or absence of 500 nM 3,4‐cPP for 24 h (D and E). *p < 0.05, **p < 0.01, ***p < 0.001, ****p < 0.0001 (one‐way ANOVA and Turkey's multiple comparison). HSPG, heparan sulfate proteoglycan; DAPI, 4′,6‐diamidino‐2‐phenylindole; CLP, cecal ligation and puncture; PBS, phosphate‐buffered saline; VE‐cad, vascular endothelial cadherin; ICAM, intercellular adhesion molecule; LPS, lipopolysaccharide; HPA1, heparinase 1.

Given the crucial role of endothelial glycocalyx in preserving vascular permeability, we examined the effect of 3,4‐cPP on CLP‐induced endothelial glycocalyx damage. CLP‐induced sepsis significantly decreased HSPG, vascular endothelial cadherin (VE‐cad), and intercellular adhesion molecule (ICAM) levels in the lungs of mice (Figures 8A–C). Meanwhile, 3,4‐cPP treatment significantly blocked the CLP‐induced decrease in HSPG, VE‐cad, and ICAM expression (Figure 8A–C).

Heparinase 1 (HPA1) is an enzyme that degrades heparan sulfate, a major component of the endothelial glycocalyx, disrupts its integrity, and leads to endothelial dysfunction [13]. We evaluated whether 3,4‐cPP affects HPA1 expression in LPS‐stimulated MYSECs. LPS increased HPA1 expression in MYSECs twofold compared with treatment with vehicle (Figure 8D). In contrast, 3,4‐cPP significantly suppressed LPS‐induced HPA1 expression (Figure 8D). SIRT1 knockdown using *Sirt1* siRNA abolished this inhibitory effect of 3,4‐cPP (Figure 8D).

The endothelial glycocalyx is sensitive to shear stress, the frictional force of blood flow [37, 38]. The expression of syndean‐2, a HSPG, is remarkably increased by shear stress [38]. Thus, we examined the effect of 3,4‐cPP on the expression of syndean‐2 in MYSECs culture under shear stress. LPS decreased syndecan‐2 expression in MYSECs cultured under shear stress compared with treatment with vehicle (Figure 8E). 3,4‐cPP restored LPS‐induced decrease in syndecan‐2 expression (Figure 8E). SIRT1 knockdown abrogated the restorative effect of 3,4‐cPP on syndecan‐2 expression (Figure 8E).

These results suggest that 3,4‐cPP protects against endothelial cell injury in sepsis by preventing HSPG degradation through SIRT1‐mediated inhibition of HPA1. Collectively, 3,4‐cPP maintains endothelial integrity via the SIRT1/HPA‐1/HSPG pathway, mitigating sepsis.

## 3 Discussion

Attempts to treat sepsis by controlling inflammation have not been successful, with no effective drug available. Recent studies on sepsis indicate that treatments must manage inflammation and strengthen the blood vessel wall [39, 40]. We previously identified SIRT1 as a key target for sepsis treatment, capable of controlling inflammation and strengthening the blood vessel wall [30, 31]. Furthermore, IFN‐β, which induces SIRT1, offers protection against sepsis [30, 31]. Although IFN‐β treatment succeed in a phase II trial for acute respiratory distress syndrome (ARDS), a severe complication of sepsis, it failed in phase III due to a polymorphism in the glucocorticoid receptor‐binding motif of IFN‐α/β receptor (IFNAR) β chain (IFNAR2) gene, affecting IFNAR expression in response to glucocorticoids [41]. This suggests that other inducers of SIRT1 expression could be effective in ARDS and sepsis. Here, we demonstrate that 3,4‐cPP, a SIRT1 inducer, effectively protects against sepsis by reducing vascular leakage and regulating inflammatory responses.

S1P plays a vital role in immune cell trafficking, inflammation, and endothelial integrity [42]. Additionally, S1P regulates the pathophysiological processes of sepsis, including increased endothelial cell permeability [43]. Patients with sepsis have reduced serum S1P, while patients who recover have higher S1P levels than those who die [21, 44]. S1P concentration is also reduced in the blood of COVID‐19 patients and correlated with disease severity [45]. A low plasma S1P concentration is a marker of worse prognosis in COVID‐19 patients [42]. Hence, S1P1 agonists and S1P2 antagonists could provide a new strategy for managing COVID‐19 by regulating the exaggerated inflammatory response against SARS‐CoV‐2 infection and the related endothelial dysfunction and stimulated inflammatory signaling pathways [19]. These results suggest that increasing serum S1P concentration via intravenous injection can treat sepsis. However, administrating exogenous S1P to increase the serum concentration is difficult due to its short half‐life (*t*1/2) (approximately 15 min) [23]. Additionally, increasing S1P concentrations can disrupt endothelial barrier function via S1P2 and S1P3, while enhancing it via S1P1. Similarly, S1P inhibits acute lung injury (ALI) via S1P1, whereas S1P2 causes ALI and pulmonary edema [46]. Thus, the exogenous administration of S1P is unsuitable for treating sepsis. In contrast, 3,4‐cPP exhibited excellent plasma stability, bound to and activated S1P1 and exerted remarkable effects on the survival of septic mice.

Fingolimod is an immunomodulatory drug that binds S1P1, S1P3, S1P4, and S1P4 to treat relapsing‐remitting multiple sclerosis [47]. Fingolimod binds, internalizes, and downregulates S1P1, which regulates lymphocyte exit from the thymus and peripheral lymphoid organs [48]. This downregulation reduces autoaggressive lymphocyte infiltration into the central nervous system and exerts therapeutic effects [48]. We also observed that fingolimod downregulated S1P1 expression by inducing internalization in macrophages, indicating that it acts as a functional antagonist rather than an agonist. In contrast, 3,4‐cPP activated S1P1, indicating that it functions as an activator that induces negligible levels of S1P1 internalization. In addition, the BioMAP assay revealed different mechanism of action for 3,4‐cPP and fingolimod. Moreover, fingolimod was ineffective against sepsis at the effective dose of 3,4‐cPP. This further demonstrates the unique biological effects of 3,4‐cPP and fingolimod due to differences in activation and internalization coupling. More specifically, fingolimod and most reported S1P1 agonists are functional antagonists that decrease cell surface S1P1 expression [49]. They can significantly inhibit lymphocyte egress from lymphoid organs and induce lymphopenia, promoting their therapeutic effects in several autoimmune diseases [22]. However, our results indicate that 3,4‐cPP is a functional agonist that activates S1P1 without internalization and suppresses the inflammatory response through the S1P1/SIRT1 pathway without inducing lymphopenia.

Transcription factors such as CREB, FOXO1, FOXO3a, p53, and NF‐kB regulate SIRT1 gene expression [50, 51, 52]. Meanwhile, SIRT1 protein expression is nontranscriptionally increased by stabilization via ubiquitin hydrolase (USP22 and USP7) activation [53]. 3,4‐cPP increased SIRT1 expression in a transcription‐dependent manner in RAW cells and a transcription‐independent manner in MYSECs. This suggests that 3,4‐cPP increases SIRT1 expression by activating transcription factors in RAW cells and activating ubiquitin hydrolases in MYSECs.

This study had certain limitations. First, although we found that 3,4‐cPP is highly effective against CLP‐induced sepsis, its effective dose range is relatively small, with minimal effects at doses approximately five times less than or greater than the optimal and a decrease in effectiveness at doses approximately five times greater than the optimal dose. Second, 3,4‐cPP may have targets other than S1P1. Further studies are needed to determine whether the narrow effective dosage range is due to the off‐target effects of 3,4‐cPP. In serum, S1P binds to high‐density lipoprotein (HDL) or albumin and transports them to its site of action [54]. Similarly structured 3,4‐cPP also appears to bind HDL and albumin and act on S1P1. Therefore, the significantly reduced concentrations of HDL and albumin in sepsis may reduce the *t*1/2 in serum and exacerbate the off‐target effects of 3,4‐cPP. To circumvent this, formulations capable of stabilizing blood 3, 4‐cPP levels during sepsis are needed.

Cumulatively, the finding of this study suggests that 3,4‐cPP upregulates SIRT1 by continuously activating S1P1, suppressing proinflammatory immune responses and preventing endothelial cell damage, protecting against sepsis. Sustained activators of S1P1 and SIRT1 may be important targets for sepsis therapeutics. Thus, 3,4‐cPP, an S1P1/SIRT1 activator, is a potential candidate for treating sepsis and related complications, including ARDS. Further research is needed to explore its therapeutic potential in human sepsis.

## 4 Materials and Methods

### 4.1 Synthesis of 3,4‐cPP

3,4‐cPP was synthesized using the following process: from commercially available phytosphingosine, amine group protection with Boc_2_O and primary hydroxyl group protection with tert‐butyldimethylsilyl chloride (TBDMS) was performed to afford the N‐Boc‐1‐TBDMS–phytosphingosine intermediate, which was cyclized with POCl_3_ under the condition of a pyridine base. The obtained intermediate, 1‐TBDMS‐2‐N‐Boc 3,4‐cPP, was reacted stepwise with HF/pyridines to deprotect 1‐TBDMS and HCl for deprotection of N‐Boc to give the final target molecule 3,4‐cPP:

^1^H NMR (400 MHz, CD_3_OD): *δ* 0.88–0.91(3H, t), 1.51–1.57(1H, m), 1.82–1.87(1H, m), 3.61–3.62(1H,), 3.89–3.96(2H, m), 4.09–4.18(1H,), 4.42–4.49(1H).
^13^C NMR (400 MHz, CD_3_OD) : *δ* 14.47, 23.77, 25.80, 30.52, 30.85, 33.11, 34.60, 48.36, 48.57, 48.79, 49.00, 49.22, 49.43, 49.64, 50.01, 65.45, 73.08, 80.38. ES–MS *m*/*z*: 380.45 [M+H]^+^

### 4.2 Agonist Activity of 3,4‐cPP Toward S1P1–4

The agonist activity of 3,4‐cPP toward S1P1–4 in PathHunter Chinese hamster ovary (CHO)‐K1 EDG1, EDG3, EDG5, and EDG6 β‐arrestin cell lines (coexpressing ProLink‐tagged human S1P1–4 and enzyme acceptor‐tagged β‐arrestin) was determined using Eurofins DiscoverX (Freemont, CA, USA) by a PathHunter detection kit.

### 4.3 The BioMAP® Phenotypic Profiling Assay

The BioMAP Diversity PLUS assay was performed using the Eurofins DiscoverX platform. The cell types, stimuli, and biomarker readouts of the BioMAP Diversity PLUS panel are presented in Figure S2. Detailed methods were described in Shah et al. [55].

### 4.4 Molecular Docking Studies

Blind docking was performed using the AutoDock program in PyRx‐Python 0.8. The X‐ray crystal structure of the S1P1 complex with S1P was retrieved from the Protein Database (https://www.rcsb.org/) in the PDB format. 3,4‐cPP was converted to PDB file format using Discovery Studio Visualizer v21. Before molecular docking, energy minimization of all ligands was performed using Discovery Studio Visualizer v21. For molecular docking, S1P1 and ligand preparation were performed using AutoDock tools, and the output files were saved in pdbqt format. The postdocking S1P1–ligand complex structures were analyzed for ligand–protein binding interactions using the free Discovery Studio Visualizer for 2D and PyMOL version 2.5.5. for immersive 3D.

### 4.5 Flow Cytometry

To assess S1P1 internalization, RAW cells were cultured with 500 nM‐2 µM fingolimod and 3,4‐cPP for 1.5 h. The cells were washed and resuspended with phosphate‐buffered saline (PBS) containing 1% bovine serum albumin (BSA) and 0.1% sodium azide (FACS buffer). The resuspended cells were stained with an anti‐S1P1 antibody (1:100, MABC94; Merck, Rahway, NJ, USA) at 4°C for 40 min and then an Alexa Fluor 488 goat anti‐mouse IgG (1:100, A11029; Invitrogen, Carlsbad, CA, USA) for 30 min at 4°C in the dark. Mouse isotypic IgG (1:100, MABC002; Merck) was used as a negative control. The cells were washed twice with FACS buffer, fixed with 0.5% paraformaldehyde, and subjected to flow cytometric analysis using a FACSCalibur flow cytometer (Becton Dickinson, Heidelberg, Germany).

### 4.6 Mice

Male C57BL/6 mice, aged 7–8 weeks and weighing between 20 and 24 g, were acquired from Nara‐Biotec in Seoul, Korea. The mice were housed in a regulated setting at 22 ± 2°C with a 12‐h light/dark cycle. Food and water were provided ad libitum. Water and food were provided ad libitum. Mice were randomly divided into the experimental or control groups (n = 10 mice/group). All animal studies received approval from the Institutional Animal Care and Use Committee at Jeonbuk National University (JBNU‐2021‐099) and adhered to the committee guidelines.

### 4.7 CLP Surgery

CLP was carried out as previously described [31]. Briefly, the cecum was partially ligated, then punctured using an 18‐gauge needle, and gently pressed to expel a small droplet (1–2 mm) of feces. After returning the cecum to the abdominal cavity, the abdomen was stitched up. The mice received an injection of 0.5 mL of prewarmed sterile saline right after the operation. The sham group underwent the same surgical procedure without ligation or puncture.

### 4.8 In Vivo Treatment with 3,4‐cPP

3,4‐cPP was dissolved in 0.1 N NaOH to 5 mg/mL and diluted 50,000 times with PBS. Mice were intravenously injected with 3,4‐cPP (5 µg/kg) twice a day at 6 and 16 h following CLP surgery. Survival duration was monitored each day for 10 days following CLP or sham operation.

### 4.9 Generation of EC–*Sirt1* cKO and Mφ–*Sirt1* cKO

Mφ–*Sirt1* cKO and EC–*Sirt1* cKO mice were generated as previously described [31, 35]. Briefly, for EC–*Sirt1* cKO mice, *Sirt1*flox/flox mice were crossed with transgenic mice expressing Cre recombinase under the control of an EC‐specific Tek promoter (Tek‐Cre). For MФ–*Sirt1* cKO, *Sirt1*flox/flox mice crossed with transgenic mice expressing Cre recombinase under the control of an MФ‐specific lyz promoter (lyz‐Cre).

### 4.10 Cell Culture and siRNA Transfection

MYSECs (ATCC# CRL‐2581) and Raw 267.4 cells (ATCC# TIB‐71) from the American Type Culture Collection (Manassas, VA, USA) were cultured with 5% CO₂ at 37°C in RM medium (Dulbecco's modified Eagle's medium containing 10% fetal bovine serum, 0.2% glutamax, and 0.5% penicillin–streptomycin). The cell lines were authenticated by short tandem repeat (STR) profiling (Table S1) and confirmed to be mycoplasma free. RAW cell line was authenticated by mouse cell line authentication service (Applied Biological Materials, Richmond, BC, Canada) using STR. MYSEC cell line was authenticated by ATCC cell line authentication service (ATCC, Manassas, VA, USA) using STR.

Negative control siRNA (Medium GC) and *Sirt1*‐siRNA (MSS203772 of Stealth siRNA) were purchased from Invitrogen and transfected at 50 nM with a transfection reagent (Invitrogen) mixed with siRNA Transfection Medium (Invitrogen).

### 4.11 MYSEC Culture Under Shear Stress

Confluent MYSECs cultured in a μ‐Slide I 0.4 chamber (ibidi GmbH, Germany) using RM medium were exposed to unidirectional laminar flow with a pumping system (Ismatec, Japan) at 37°C in a humid environment with 5% CO_2_. 10 dyn/cm^2^ Laminar shear stress was applied for 24 h. Subsequently, 500 nM 3,4‐cPP was added to the flow medium in the presence of 300 ng/mL LPS. SIRT1 in MYSECs were knockdown by transfection with *Sirt1* siRNA 48 h prior to plating and flow initiation.

### 4.12 Western Blotting

Cell lysates (20 µg) prepared using Thermo Fisher Scientific T‐PER tissue protein extraction reagent (Waltham, MA, USA) were fractioned through 10% sodium dodecyl sulfate‐polyacrylamide gel electrophoresis (SDS‐PAGE) and then transferred onto polyvinylidene fluoride membranes. The membranes were blocked with Tris‐buffered saline (pH 7.4) containing 3% nonfat milk and 0.1% Tween 20 for 1 h at room temperature. They were then incubated overnight at 4°C with primary antibodies targeting SIRT1 (1:1000, B‐7 sc‐74465; Santa Cruz Biotechnology, Dallas, TX, USA), HPA1 (1:500, ab228660; Abcam, Cambridge, UK), and β‐actin (1:5000, A5441; Sigma, St. Louis, MA, USA). After washing, the membranes were treated with horseradish peroxidase (HRP) conjugated secondary antibodies at room temperature for 1 h. Protein bands were detected with the Fusion Fx7 Spectra (Vilber Lourmat, Collégien, France). The FusionCapt software version 16.08 (Vilber Lourmat Sté, Collégien, France) was utilized to quantify protein expression.

### 4.13 Histopathological Evaluation of Tissue Injury

Eighteen hours after CLP surgery, the mouse organs were collected and immediately fixed with 10% paraformaldehyde at 4°C for 24 h. The samples were then embedded in paraffin, transversely sliced into 5 µm‐thick sections, and deparaffinized with xylene. Finally, the slides were H&E‐stained for histological evaluation under a light microscope at a 400× magnification. Organ injury severity was scored using semiquantitative scoring systems as previously described [56, 57]. Three symptoms (edema, inflammation, and hemorrhage) in the lung sections were scored on a 0–4 scale based on damage severity. Similarly, three symptoms (interstitial edema, inflammation, and erythrocyte stasis) in the kidney sections were scored a 0–4 scale based on damage severity. Meanwhile, three symptoms (inflammation, coagulation, and lipid accumulation) in the liver sections were scored a 0–3 scale based on damage severity. The total sums of each symptom score were recorded as the obtained organ damage scores.

### 4.14 In Vitro Vascular Permeability Assays

MYSECs (2.5 × 10^5^ cells per well) were grown in RM medium on the upper chamber of 0.4 µm Transwell inserts (Corning B.V. Life Sciences, The Netherlands) with 300 ng/mL LPS for 24 h in the presence of 500 nM 3,4‐cPP. The medium in the upper chambers was replaced with 300 µL of serum‐free medium containing 5 µL of streptavidin‐HRP (#DY998; R&D Systems, Minneapolis, MN, USA). After 5 min, 20 µL of the medium from the lower chambers was transferred to new 96‐well plates and HRP activity was measured by adding 100 µL of stabilized hydrogen peroxide and 3,3′,5,5′‐tetramethylbenzidine solution (#DY999; R&D Systems). Using a Molecular Devices microplate reader (Sunnyvale, CA, USA), the absorbance at 450 nm was measured.

### 4.15 In Vivo Vascular Permeability Assay

Vascular permeability was assessed through the extravasation of EBD (Sigma–Aldrich). The mice received an injection of 200 µL 0.5% EBD via the caudal vein at 18 h post‐CLP surgery. The organs were removed and imaged after allowing EBD to circulate for 1 h. The organs were then dried with blotting paper, weighed, and blended in formamide (Sigma–Aldrich). The blending mixture were incubated for 48 h at 55°C and then centrifuged for 25 min at 12,000×*g*. A Molecular Devices microplate reader (Sunnyvale, CA, USA) was utilized to evaluate EBD level in the supernatant at 620 nm.

### 4.16 Tissue Wet‐to‐Dry Weight Ratio

The kidneys, lungs, and liver were weighed immediately after removal (wet weight) and were placed in an oven at 60°C for 48 h. The dried tissue portions were weighed (dry weight). Kidney, lung, and liver weight ratios were calculated before and after drying. Tissue wet‐to‐dry weight ratio was used as an index of tissue edema formation.

### 4.17 Real‐Time Polymerase Chain Reaction

In real‐time polymerase chain reaction (RT‐PCR) analysis, the templates were amplified with the Superscript III cDNA amplification system (Invitrogen) in a thermocycler (ABI Prism 7,000; Applied Biosystems). The following primers were used for each mouse gene: *Gapdh* forward 5′‐CATCACTGCCACC CAGAAGACTG‐3′, reverse 5′‐ATGCCAGTGAGCTTCCCGTTCAG‐3′; *Sirt1* forward 5′‐TCGTGGAGACATTTTTAATCAGG‐3′, reverse 5′‐GCTTCATGATGGCAAGTGG ‐3′. The data were analyzed using the ABI Prism 7,000 SDS Software and expressed using the 2^−ΔΔCt^ method.

### 4.18 Immunofluorescence

Lung tissue sections were blocked with PBS containing 5% BSA, 5% goat serum, and 0.05% Tween‐20 at 25°C for 1 h. The sections were incubated with anti‐HSPG antibody (1:25, MA1‐06821; Invitrogen) overnight at 4°C. After washing, the samples were treated with a secondary antibody conjugated to fluorescein isothiocyanate (Invitrogen) at 37°C for 1 h. Finally, the samples were stained with 4′,6‐diamidino‐2‐phenylindole (DAPI) as a counter stain.

### 4.19 ELISA

The levels of TNF‐α and IL‐6 were measured using ELISA kits (Koma Biotechnology, Seoul, South Korea). Eighteen hours after CLP administration, the mice were anesthetized, and blood samples were collected via facial vein puncture and stored at room temperature for 1 h. The serum or cell supernatant was separated by centrifugation at 3000 rpm or 800×*g* for 10 min. The levels of TNF‐α and IL‐6 were measured according to the manufacturer's instructions.

### 4.20 Statistical Analysis

Survival curves were plotted using the Kaplan–Meier method and assessed with the log‐rank test for comparison. One‐way analysis of variance (ANOVA) was used to compare differences between more than two groups, and *p* values < 0.05 indicated significance. Statistical assessments were conducted with GraphPad Prism 5.0 (GraphPad Software Inc., San Diego, CA, USA).

## Author Contributions

Suhong Duan: Formal analysis, investigation, validation, and writing of original draft. Seung‐Gook Kim: Formal analysis, investigation, and validation. Bao Jiyang: Formal analysis, investigation, and validation. Hyung‐Jin Lim: Formal analysis, investigation, and validation. Joon Woo Kim: Formal analysis and investigation. Sung‐IL Yoon: Formal analysis and investigation. Young Jun Park: Formal analysis and investigation. Sanuk Yun: Investigation, data curation, and validation. Kye‐Seong Kim: Investigation, data curation, and validation. Hwa‐Ryung Song: Project administration, data curation, validation, and supervision. Myeong Jun Choi: project administration, data curation, and validation. Myung‐Kwan Han: Conceptualization, project administration, funding acquisition, writing, review, and editing. All the authors have read and approved the final manuscript.

## Ethics Statement

All animal studies were approved from the Institutional Animal Care and Use Committee at Jeonbuk National University (JBNU‐2021‐099) and adhered to the committee guidelines.

## Conflicts of Interest

Joon Woo Kim, Sung‐Il Yoon, Young Jun Park, and Myeong Jun Choi are employees in Axceso Biopharma, but have no potential relevant financial or nonfinancial interests to disclose. The other authors declare no conflicts of interest.

## Supporting information



Supporting information

## Data Availability

All relevant data related to this study, whether produced or analyzed, are thoroughly included in this manuscript and its Supporting Information. Those who are interested are invited to reach out to the corresponding authors for any further questions or requests.
