# Supplementary material for: Persistent Activation of Sphingosine‐1‐Phosphate Receptor 1 by Phytosphingosine‐3,4‐Cyclic Phosphate Ameliorates Sepsis by Inhibiting Hyperinflammation and Vascular Hyperpermeability
Source: MedComm (2020). 2025 Jun 3;6(6):e70238. doi: 10.1002/mco2.70238 (PMC12134398; doi:10.1002/mco2.70238)
Supplement: Supplementary file 1 — Supporting information [file MCO2-6-e70238-s001.docx]

**Supporting Information**

Persistent activation of S1P1 by phytosphingosine-3,4-cyclic phosphate ameliorates sepsis by inhibiting hyperinflammation and vascular hyperpermeability

Suhong Duan^1,2,#^, Seung-Gook Kim^1,#^, Jiaying Bao^1^, Hyung-Jin Lim^1^, Joon Woo Kim^3^, Sung-Il Yoon^3^, Young Jun Park^3^, Sanuk Yun^4^, Kye-Seong Kim^5^, Hwa-Ryung Song^1,*^, Myeong Jun Choi^3,*^ and Myung-Kwan Han^1,*^

^1^Department of Microbiology, Jeonbuk National University Medical School, 565 Baekje-daero, Jeonju-si, Jeollabuk-do 54896, Republic of Korea

^2^Department of Pediatrics, Shandong Provincial Hospital Affiliated to Shandong First Medical University, Jinan, Shandong province, 250021, China

^3^Axceso Biopharma, 282 Hagui-ro, Yongin-si, Gyeonggi-do 16914, Republic of Korea

^4^Department of Biotechnology, Inje University, Gimhae, 50834, Republic of Korea

^5^Graduate School of Biomedical Science and Engineering, Hanyang University, 222 Wangsimni-ro, Seongdong-gu, Seoul 04763, Republic of Korea

***Corresponding authors**

Hwa-Ryung Song and Myung-Kwan Han, Department of Microbiology, Jeonbuk National University Medical School, Jeonju 54896, Republic of Korea.

E-mail: silverysk@hanmail.net and iamtom@chonbuk.ac.kr

Myeong Jun Choi, Axceso Biopharma, Yongin-si, Gyeonggi-do16914, Republic of Korea.

E-mail: [myeongjun@gmail.com](mailto:myeongjun@gmail.com)

**Experimental Section**

***S.1. Synthetic chemistry and NMR validation of structures*.** All reactions were carried out in oven-dried glassware under a nitrogen atmosphere with freshly distilled dry solvents under anhydrous conditions unless otherwise indicated. Flash column chromatography was performed using Silica Flash P60 silica gel (230–400 mesh). All reagents were obtained from commercial sources and used without further purification. Proton (1H) and carbon (13C) nuclear magnetic resonance (NMR) spectra were recorded on a JEOL 400 MHz FT-NMR spectrometer. NMR solvents were obtained from Cambridge Isotope Laboratories, and the residual solvent signals were used as the reference (CD3OD, 3.31 ppm for 1H NMR spectra and CD3OD 49.03 ppm for 13C NMR spectra). Mass analysis was performed using a Waters LC/3100 mass spectrometer.

***S.2. Synthesis of phytosphingosine-3,4-cyclic phosphate (3,4-cPP)***

**----------------------------------------------------------------------------------------------------------------**

***Step 1: tert-butyl ((2S,3S,4R)-1,3,4-trihydroxyoctadecan-2-yl)carbamate (2).*** Boc_2_O (8.3 g, 37.794 mmol) was added to a solution of DS-phytosphingosine (10 g, 31.495 mmol) in tetrahydrofuran (100 mL) at RT. The mixture was stirred for 18 h at RT and then concentrated in vacuo. The crude material was purified by crystallization with hexane (80 mL) to yield **2** (11.8 g, 28.255 mmol) as a white solid.

^1^H NMR (400 MHz, CD_3_OD) : δ 0.88-0.91(3H, t), 1.29(24H, m), 1.44(9H, s), 1.53-1.72(2H, m), 3.53-3.54(2H, m), 3.64-3.69(1H, m), 3.71-3.77(2H, m)

^13^C NMR (400 MHz, CD_3_OD) : δ 14.44, 23.74, 27.05, 28.79, 30.47, 30,76, 30.78, 30.80, 33.07, 33.19, 48.36, 48.57, 48.78, 49.00, 49.21, 49.43, 49.64, 54.58, 62.46, 73.35, 76.51, 80.19, 157.95

Electrospray ionization mass spectrometry (ES-MS) mass-to-charge ratio (m/z): 418.44 [M+H]^+^

***Step 2: tert-Butyl ((2S,3S,4R)-1-((tert-butyldimethylsilyl)oxy)-3,4-dihydroxyoctadecan-2-yl)carbamate (3).*** A solution of tert-butyldimethylsilyl chloride (2 g, 13.170 mmol) in DCM (10 mL) was added dropwise to a solution of **2** (5 g, 11.973 mmol), and triethylamine (1.85 mL, 13.170 mmol), 4-dimethylaminopyridine (0.73 g, 5.986 mmol) in DCM (50 mL) at RT. The mixture was stirred for 16 h, and the reaction was quenched with water (30 mL). The aqueous layer was extracted with DCM and the combined organic layers were dried over Na_2_SO_4_, filtered, and concentrated in vacuo. The crude material was purified by flash chromatography to yield **3** (6.33 g, 11.901 mmol) as a clear oil.

^1^H NMR (400 MHz, CD_3_OD) : δ 0.09(6H, s), 0.88-0.91(12H, m), 1.29824H, m), 1.43(9H, s), 1.54-1.68(2H, m), 3.50-3.55(2H, m), 3.73-3.74(2H, m), 3.82-3.87(1H, m)

^13^C NMR (400 MHz, CD_3_OD) : δ -5.27, 14.46, 19.16, 20.87, 23.74, 26.44, 27.01, 28.85, 30.48, 30,78, 30.80, 32.90, 33.07, 48.36, 48.57, 48.78, 49.00, 49.21, 49.43, 49.64, 54.72, 61.51, 63.76, 73.28, 76.16, 80.09, 157.76, 172.93

ES-MS m/z: 532.51 [M+H]^+^

***Step 3: tert-Butyl ((1S)-2-((tert-butyldimethylsilyl)oxy)-1-((4S,5R)-2-methoxy-2-oxido-5-tetradecyl-1,3,2-dioxaphospholan-4-yl)ethyl)carbamate (4).*** A solution of methyl phosphorodichloridate (2.9 mL, 24.441 mmol) in DCM (20 mL) was added dropwise to a solution of **3** (10 g, 18.801 mmol) and pyridine (6.13 mL, 75.205) in DCM (80 mL) at 0℃. The mixture was stirred for 1 h, slowly warmed to RT for 1 h, and stirred for 3 h. The reaction was quenched by adding water (50 mL). The aqueous layer was extracted with DCM (50 mL) and the combined organic layers were dried over Na_2_SO_4_, filtered, and concentrated in vacuo. The crude material was subjected to the following reaction, without purification:

ES-MS m/z: 608.63 [M+H]^+^

***Step 4: tert-Butyl ((1S)-2-hydroxy-1-((4S,5R)-2-methoxy-2-oxido-5-tetradecyl-1,3,2-dioxaphospholan-4-yl)ethyl)carbamate (5).*** A solution of hydrogen fluoride pyridine (0.64 mL, 24.675 mmol) was added to a solution of **4** (3 g, 4.935 mmol) in Tetrahydrofuran (30 mL) at RT. The mixture was then stirred for 16 h, and the reaction was quenched with saturated NaHCO_3_ solution (10 mL) diluted in water. The aqueous layer was extracted with ethyl acetate and the combined organic layers were dried over Na_2_SO_4_, filtered, and concentrated in vacuo. The crude material was purified by flash chromatography to yield **5** (1.73 g, 3.504 mmol) as a clear oil.

Rf=0.09 (EA:Hexane = 1:1)

^1^H NMR (400 MHz, CD_3_OD) : δ 0.88-0.91(3H, t), 1.29(24H, m), 1.45(9H, s), 1.54-1.72(2H, m), 3.77-3.83(4H, m), 4.09-4.30(3H, m), 4.41-4.47(1H, m)

^13^C NMR (400 MHz, CD_3_OD) : δ 14.44, 23.74, 26.61, 28.68, 30.52, 30.70, 30.74, 30.78, 32.87, 33.07, 46.85, 48.36, 48.57, 48.79, 49.00, 49.22, 49.43, 49.64, 55.64, 69.99, 71.24, 80.92, 87.29, 157.36

ES-MS m/z: 494.42 [M+H]^+^

Rf=0.19 (EA:Hexane = 1:1)

^1^H NMR (400 MHz, CD_3_OD) : δ 0.88-0.91(3H, t), 1.29(24H, m), 1.43(9H, s), 1.54-1.63(2H, m), 3.71-3.73(1H, m), 3.80-3.84(3H, d), 4.00-4.01(1H, m), 4.06-4.12(1H, m), 4.20-4.28(1H, m), 4.32-4.36(1H, m)

^13^C NMR (400 MHz, CD_3_OD) : δ 14.45, 23.74, 26.96, 28.66, 30.47, 30,74, 30.76, 30.80, 33.07, 46.38, 48.36, 48.58, 48.79, 49.00, 49.13, 49.22, 49.43, 49.64, 54.75, 70.02, 71.45, 81.06, 86.67, 157.11

ES-MS m/z: 494.42 [M+H]^+^

***Step 5: tert-Butyl ((1S)-2-hydroxy-1-((4S,5R)-2-hydroxy-2-oxido-5-tetradecyl-1,3,2-dioxaphospholan-4-yl)ethyl)carbamate t-butylamine salt (6).*** A solution of **5** (1.37 g, 2.775 mmol) in tert-butylamine (20 mL) was stirred at 80 °C for 13 h and cooled to RT. The mixture was then diluted with ethyl acetate (20 mL) and filtered. The crystals were vacuum dried to obtain **6** (1.15 g, 2.081 mmol) as a white solid.

^1^H NMR (400 MHz, CD_3_OD) : δ 0.88-0.91(3H, t), 1.29(24H, m), 1.35(9H, s), 1.43(9H, s), 1.47-1.70(2H, m), 3.68-3.71(1H, m), 3.89-4.11(4H, m)

^13^C NMR (400 MHz, CD_3_OD) : δ 14.47, 23.76, 27.08, 27.71, 28.72, 30.51, 30,66, 30.84, 31.87, 33.10, 47.58, 48.36, 48.57, 48.79, 49.00, 49.22, 49.43, 49.64, 52.58, 68.47, 72.52, 80.47, 83.42, 157.50

ES-MS m/z: 480.52 [M+H]^+^

***Step 6: (4S,5R)-4-((S)-1-amino-2-hydroxyethyl)-2-hydroxy-5-tetradecyl-1,3,2-dioxaphospholane 2-oxide hydrochloride (1).*** A solution of 4M HCl in dioxane (11 mL, 41.62 mmol) was added to a solution of **6** (1.15 g, 2.081 mmol) in ethyl acetate (20 mL) at RT. The mixture was stirred for 5 h and filtered. The crystals were washed with water and vacuum-dried to obtain **1** (0.69 g, 1.665 mmol) as a white solid.

^1^H NMR (400 MHz, CD_3_OD) : δ 0.88-0.91(3H, t), 1.51-1.57(1H, m), 1.82-1.87(1H, m), 3.61-3.62(1H, ), 3.89-3.96(2H, m), 4.09-4.18(1H, ), 4.42-4.49(1H, )

^13^C NMR (400 MHz, CD_3_OD) : δ 14.47, 23.77, 25.80, 30.52, 30.85, 33.11, 34.60, 48.36, 48.57, 48.79, 49.00, 49.22, 49.43, 49.64, 50.01, 65.45, 73.08, 80.38

ES-MS m/z: 380.45 [M+H]^+^

**
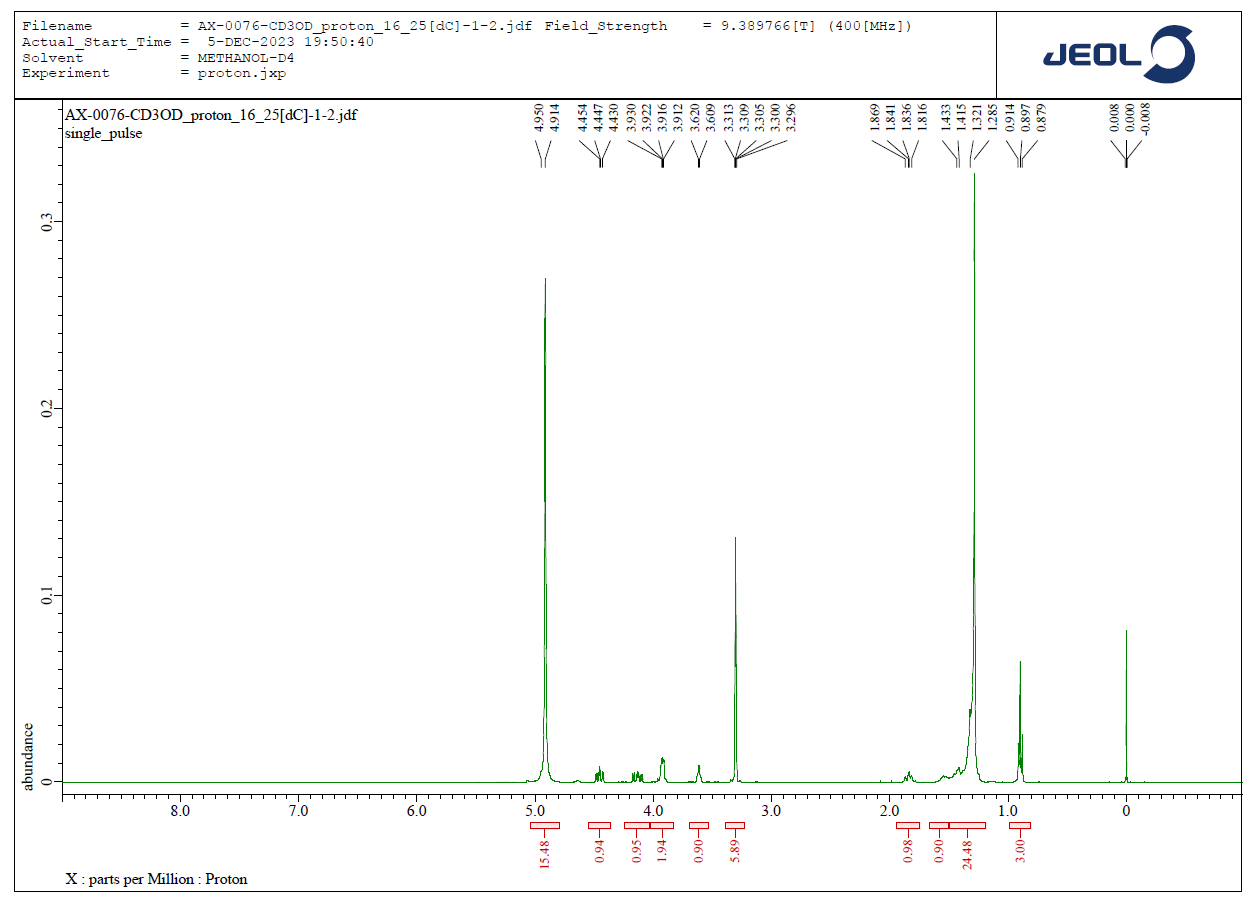
**

**
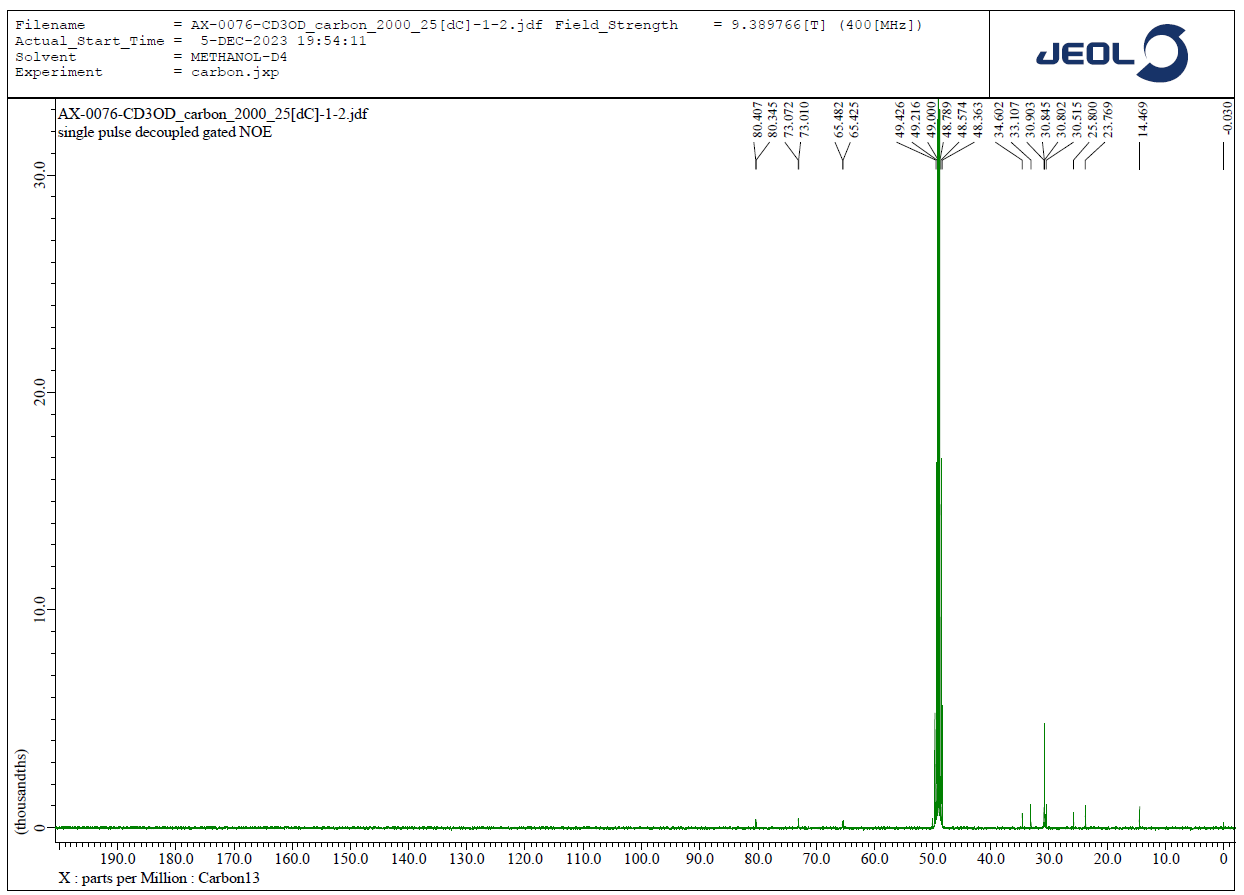
**

***S.3. Plasma stability of 3,4-cPP****.* 3,4-cPP (final incubation concentration = 2000 ng/ml) was incubated with mouse and human plasma at 37 °C for eight time points (0, 15, 30, 45, 60, 90, 120, and 180 min). Twenty microliters of the reaction mixture were transferred into a microtube and mixed with 20 µL of the internal working solution (2.5 µg/mL, dasatinib) and 200 µL methanol at 25°C, vortexed for 10 s, and then centrifuged at 10,000 × g for 5 min. An aliquot (2 µL) of the supernatant was injected into an LC-MS/MS system (Waters ACQUITY UPLC and Waters Xevo T-QS Micro, Waters, Milford, MA, USA) for 3,4-cPP quantification. A C18 chromatographic column (Acquity UPLC BEH shield RP18, 2.1 mm × 100 mm, 1.7 µm) was used for chromatographic separation. Data acquisition and processing were performed using MassLynx V4.2. (Waters): mobile phases of 0.01% acetic acid (mobile phase A) and methanol (mobile phase B); flow rate of 300 µL/min; running time per injection of 3.0 min. The mass spectrometer was operated in negative ion mode (ESl-) with an electrospray voltage of 2.15 kV and a capillary temperature of 450 °C. Nitrogen was used as desolvation gas (550 L/h) and cone gas (30 L/h). Multiple reaction monitoring (MRM) was used for quantitative analysis. The precursors were optimized for product ions and collision energies at m/z 378.3 🡪 79.01 for 3,4-cPP and m/z 486.2 🡪 261.2 for the internal standard dasatinib.

***S.4. Mice.*** Male C57BL/6 mice, aged seven to eight weeks and weighing between 20 and 24 g, were acquired from Nara-Biotec in Seoul, Korea. The mice were housed in a regulated setting at a temperature of 22 ± 2°C with a 12-h cycle of light and darkness. Food and water were provided *ad libitum*. Water and food were provided ad libitum. All animal studies received approval from the Institutional Animal Care and Use Committee at Jeonbuk National University (JBNU-2021-099) and adhered to the committee guidelines. In the in vivo experiments, mice were randomly divided into either the experimental group or the control group, each consisting of 10 mice.

***S.5. Cecal ligation and puncture (CLP).*** CLP was performed after the mice were anesthetized with 150 mg/kg ketamine and 17.5 mg/kg rompun. A 1–2 cm incision was created along the ventral midline of the abdomen on the cleaned and shaved skin. The cecum was subsequently revealed, tied with a 6-0 silk suture immediately beyond the ileocecal valve to avoid intestinal obstruction, and punctured using an 18-gauge needle. The punctured cecum was carefully pressed to release a small droplet of feces measuring 1-2 mm, which was then placed back into the abdominal cavity. The abdomen was sutured, and the mice received an intraperitoneal injection of pre-warmed normal saline (2.5 mL per 100 g of body weight) immediately after the procedure. Sham surgery was performed as described above for CLP; however, the abdominal wall was closed after the midline incision and laparotomy.

***S.6. In Vivo treatment with 3,4-cPP.*** 3,4-cPP was dissolved in 0.1 N NaOH to 5 mg/ml and diluted 50,000 times with phosphate-buffered saline (PBS). Mice were intravenously injected with 3, 4-cPP (5 µg/kg) twice daily at 6 and 18 h after CLP surgery. Survival time was recorded daily within 10 days following CLP or sham surgery.

***S.7. Cell culture and siRNA transfection******.*** MYSECs (ATCC# CRL-2581) and Raw 267.4 cells (ATCC# TIB-71) from the American Type Culture Collection (Manassas, VA, USA) were cultured with 5% CO₂ at 37°C in Dulbecco’s modified Eagle’s medium containing 10% fetal bovine serum, 0.2% glutamax, and 0.5% penicillin-streptomycin. The cell lines were authenticated by short tandem repeat (STR) profiling and tested free of mycoplasma (Table 1). For SIRT1 knockdown, negative control siRNA (Medium GC) and *Sirt1*-siRNA (MSS203772 of Stealth siRNA) were purchased from Invitrogen (Carlsbad, CA, USA) and transfected at a concentration of 50 nM with a transfection reagent (Invitrogen) mixed with siRNA Transfection Medium (Invitrogen).

***S.8. Western blotting.*** Cell lysates (20 μg) prepared using T-PER^TM^ tissue protein extraction reagent (Thermo Fisher Scientific, Waltham, MA, USA) were electrophoresed on 7.5% sodium dodecyl sulfate-polyacrylamide gels and subsequently transferred to polyvinylidene fluoride membranes. The membranes were blocked with Tris-buffered saline (pH 7.4) containing 3% nonfat milk and 0.1% Tween 20 for 1 h at room temperature. They were then incubated overnight at 4°C with primary antibodies targeting SIRT1 (1:1000, Santa Cruz Biotechnology, B-7 sc-74465, Dallas, TX, USA), S1P1 (1:1000, Sigma-Aldrich, MABC94, St. Louis, MA, USA), and β-actin (1:5000, Sigma, A5441, St. Louis, MA, USA). After washing, the membranes were treated with horseradish peroxidase (HRP) conjugated secondary antibodies at room temperature for 1 h. Protein bands were detected with the Fusion Fx7 Spectra (Vilber Lourmat, Collégien, France). The quantification of protein expression was performed using the Fusion-Capt software (version 16.08; Vilber Lourmat).


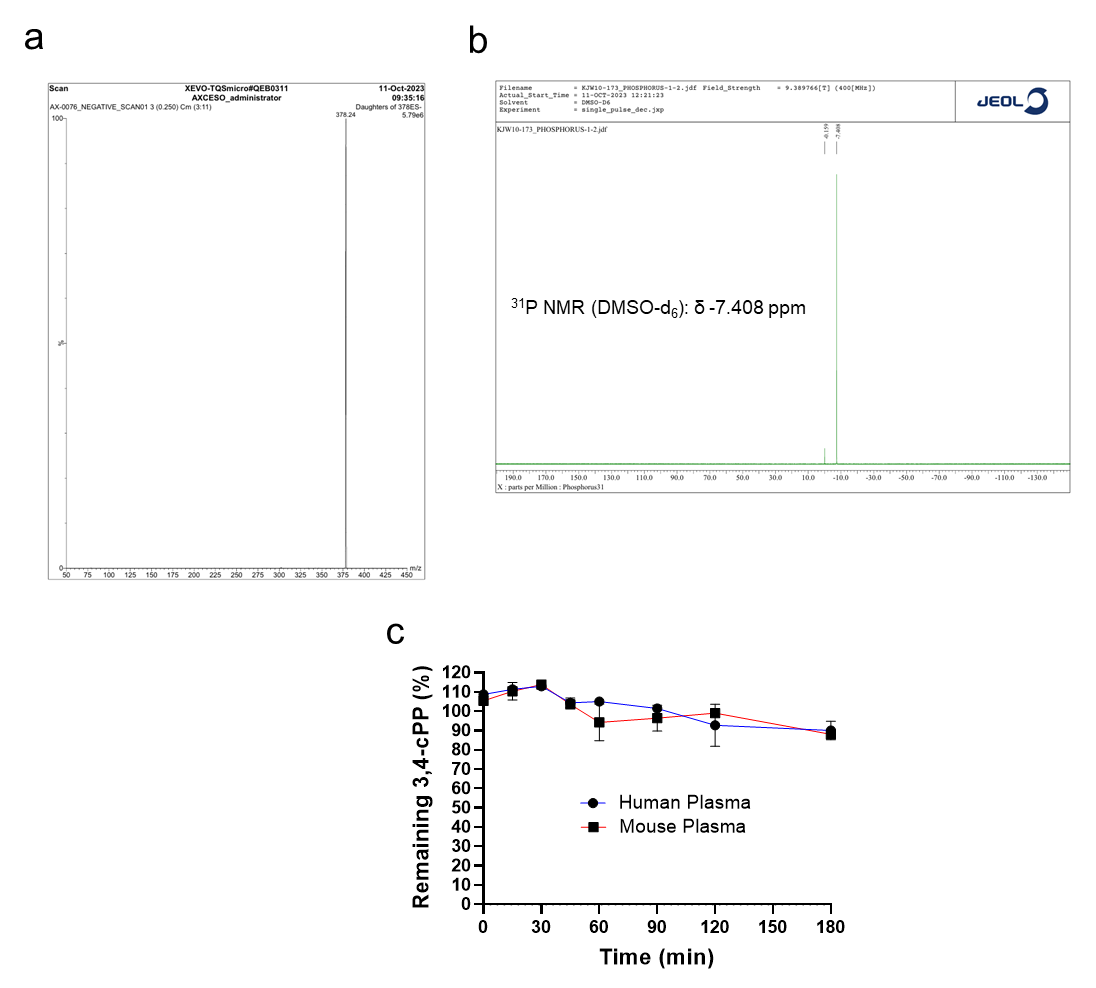


**Figure S1. Synthesis and characterization of 3,4-cPP**. (a) Representative full-scan product ion mass spectra of the [M-H] ions of 3,4-cPP. The mass spectrum measured in the negative ion mode [M-H] showed a value of 378.24, indicating that the molecular weight of 3,4-cPP as a free base is 379.47. (b) 31P NMR spectrum of 3,4-cP1P. The ^31^P NMR spectrum of 3,4-cPP shows a peak at δ -7.408 ppm. (c) Serum stability of 3,4-cPP. 3,4-cPP in mouse and human plasma was quantified using LC-MS/MS. Values represent mean ± SD from two separate experiments performed in duplicate.


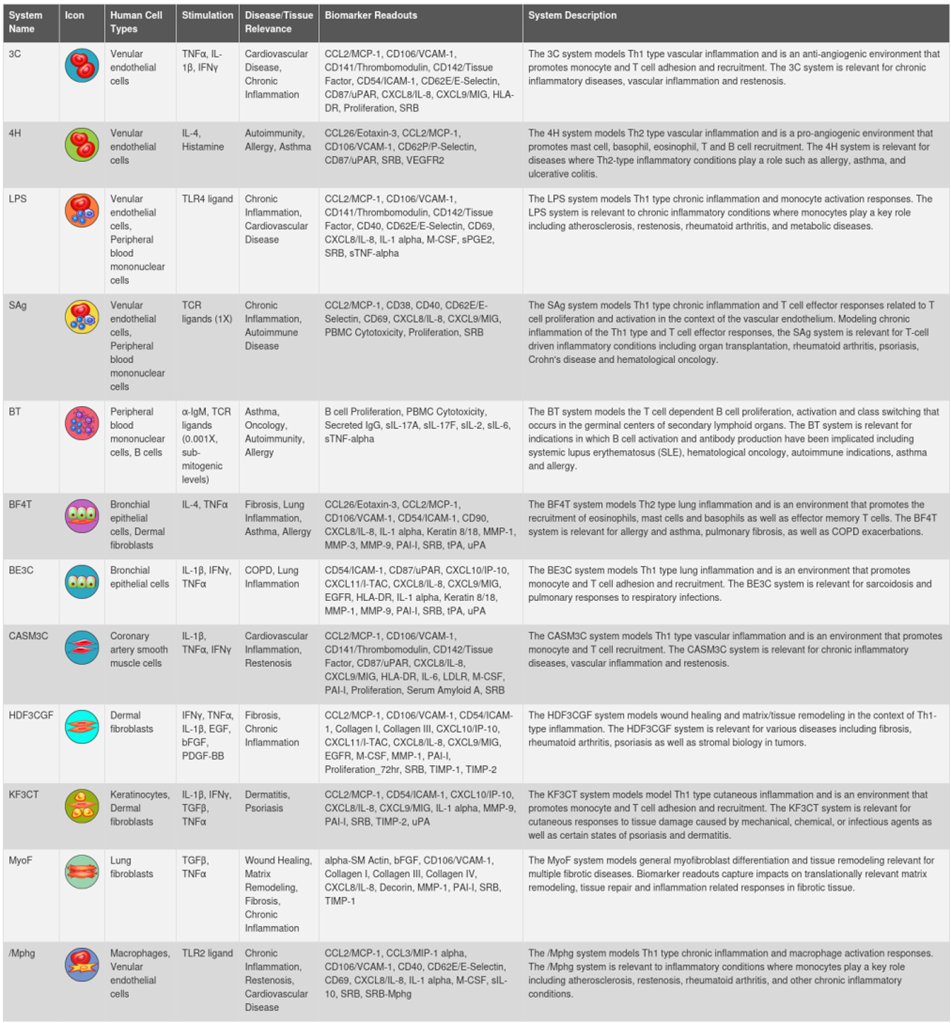


**Figure S2.** **Overview of BioMAP primary human cell systems.** IG, monokine induced by interferon γ; MMP, matrix metalloproteinase; PAI-1, plasminogen activator inhibitor-1; PBMC, peripheral blood mononuclear cell; sPGE2, soluble prostaglandin E2; sTNFα, soluble tumor necrosis factor α; TIMP, tissue inhibitor of metalloproteinases; tPA, tissue plasminogen activator; uPA, urokinase-type plasminogen activator; uPAR, urokinase receptor; VCAM-1, vascular cell adhesion molecule-1; VEGFRII, vascular endothelial growth factor receptor II.


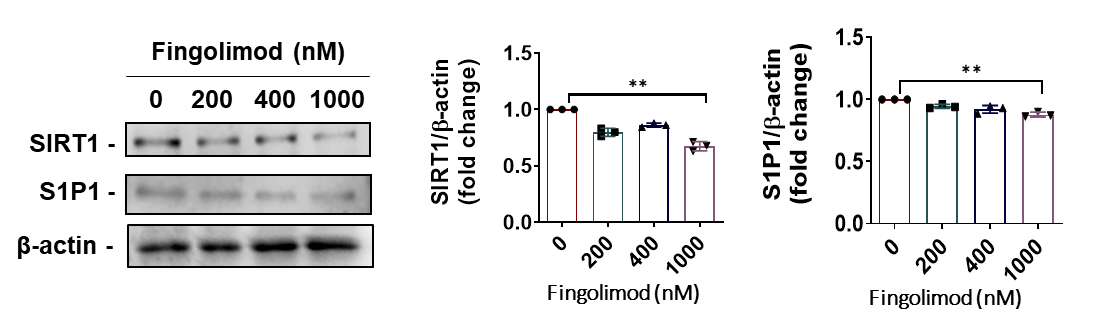


**Figure S3. Effect of fingolimod on SIRT1 and S1P1 expression in RAW 264.7 cells.** RAW 264.7 cells were treated with 0–1000 nM fingolimod for 24 h. Protein expression was analyzed using western blotting. Representative protein bands (left) and western blot quantitative analyses of SIRT1(middle) and S1P1 (right) are presented (means ± SD, n=3). *p < 0.05, **p < 0.01, ***p < 0.001, ****p < 0.0001 (one-way ANOVA and Turkey’s multiple comparison)


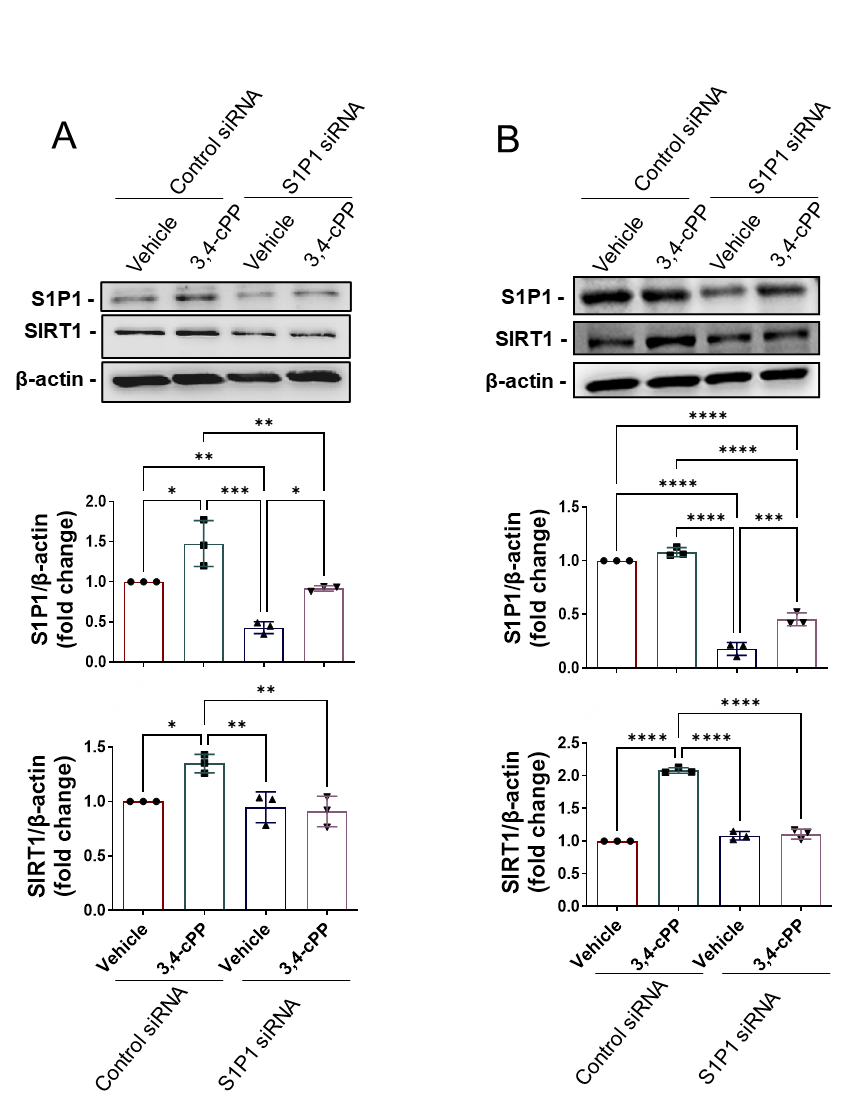


**Figure S4.** **3,4-cPP upregulates SIRT1 expression through S1P1 in RAW 264.7 cells (A) and MYSECs (B).** RAW 264.7 cells and MYSECs were transfected with control or S1P1 siRNA for 24 h and treated with 250 ng/ml 3,4-cPP for 24 h. Protein expression was analysed by western blotting. Representative protein bands (upper) and western blot quantitative analyses of S1P1 (middle) and SIRT1 (lower) are presented. Error bars represent means ± SD (n = 3 per group). **p* < 0.05, ***p* < 0.01, ****p* < 0.001, *****p* < 0.0001 (one-way ANOVA and Turkey’s multiple comparison).

**Figure S5. Intravenous fingolimod administration does not affect cecal ligation and puncture (CLP)-induced sepsis.** Fingolimod (1 and 5 µg/kg) was administered intravenously at 6 h and 16 h post-CLP. Survival was monitored for 10 days (n = 10/group).


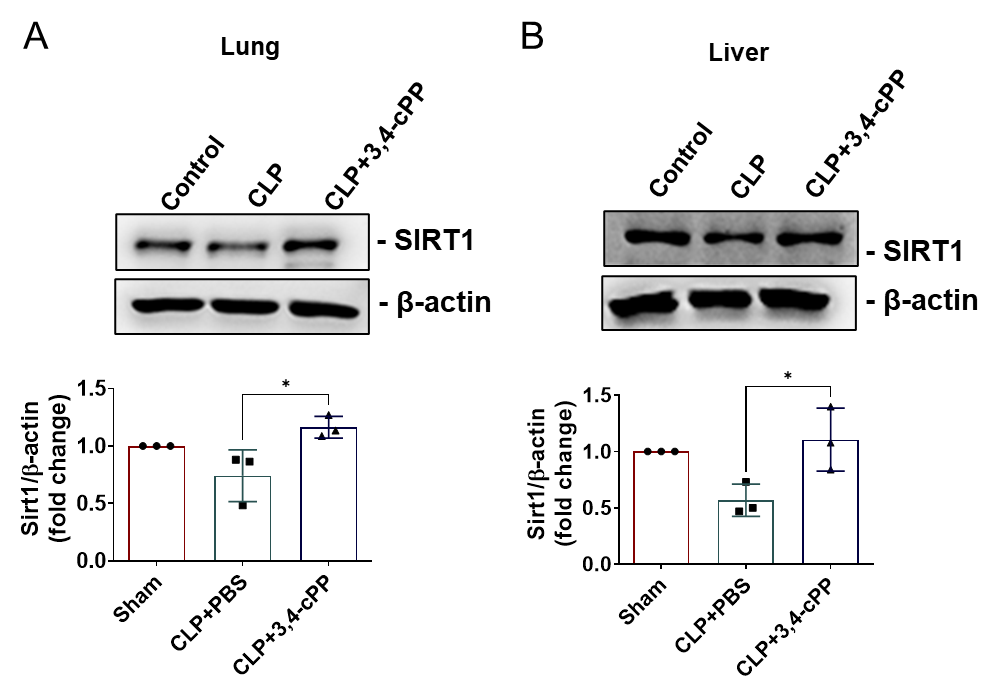


**Figure S6. 3,4-cPP increases SIRT1 expression in the lungs and liver in vivo.**

Cecal ligation and puncture (CLP) was performed on C57BL/6 mice, followed by 3,4-cPP treatment, as shown in Figure 3. Eighteen hours after the CLP operation, the lung (a) and liver (b) tissues were harvested. Protein expressions were analyzed using western blotting. Representative protein bands (uppers) and western blot quantitative SIRT1(lowers) analyses are presented. Error bars represent the means ± SD, n = 3/group. *p < 0.05 (one-way ANOVA and Turkey’s multiple comparison).

**Table S1. Results from short tandem repeat (STR) analysis of RAW and MYSEC cell lines. The STR profile matched 100% to RAW and MYSEC cell lines from American Type Culture Collection (ATCC) database which Applied Biological Materials and ATCC authenticated, respectively.**

| Marker Name | Parent RAW 264.7 (ATCC#TIB 71) | RAW cells  in this study | Parent MYSECs  (ATCC#CRL2581) | MYSECs  in this study |
| --- | --- | --- | --- | --- |
| MCA-1-1 | 15,16 | 15,16 | 10,11 | 10,11 |
| MCA-1-2 | 17 | 17 | 13 | 13 |
| MCA-2-1 | 16 | 16 | 9 | 9 |
| MCA-3-2 | 14 | 14 | 13 | 13 |
| MCA-4-2 | 22.3 | 22.3 | 19.3,20.3 | 19.3,20.3 |
| MCA-5-5 | 14 | 14 | 12,14 | 12,14 |
| MCA-6-4 | 18 | 18 | 15.3 | 15.3 |
| MCA-6-7 | 12 | 12 | 12,15 | 12,15 |
| MCA-7-1 | 25.2 | 25.2 | 25.2,29 | 25.2,29 |
| MCA-8-1 | 13 | 13 | 13,16 | 13,16 |
| MCA-9-2 | 15 | 15 | 15 | 15 |
| MCA-11-2 | 17 | 17 | 16,17 | 16,17 |
| MCA-12-1 | 16 | 16 | 16 | 16 |
| MCA-13-1 | 16.2 | 16.2 | 15 | 15 |
| MCA-15-3 | 22.3 | 22.3 | 22.3 | 22.3 |
| MCA-17-2 | 14,16 | 14,16 | 13,17 | 13,17 |
| MCA-18-3 | 18 | 18 | 17 | 17 |
| MCA-19-2 | 14 | 14 | 12 | 12 |
| MCA-X-1 | 24 | 24 | 25 | 25 |
